# Supplementary material for: Untangling the changing impact of non-pharmaceutical interventions and vaccination on European COVID-19 trajectories
Source: Nat Commun. 2022 Jun 3;13:3106. doi: 10.1038/s41467-022-30897-1 (PMC9166696; doi:10.1038/s41467-022-30897-1)
Supplement: Supplementary file 1 — Supplementary Information [file 41467_2022_30897_MOESM1_ESM.pdf]

Supplementary Information

Contents

A. Methodology 2

A1. Derivation of the instantaneous reproduction number from SIR model 2

A2. Calculation of the instantaneous basic reproduction number 3

A3. Calculation of the practical vaccination rate 6

A4. Risk of openness index 8

B. Additional results 11

B1. Effect of temperature and unknown factors 11

B2. Country-specific effect NPIs and vaccination over time 11

B3. Estimates of the coefficients 20

B4. Collinearity 24

B5. Sensitivity analysis 25

C. Model validation 27

C1. Prior and posterior predictive check 27

C2. MCMC convergence 27

C3. Meta-analysis 37

C4. Leave-one-out cross validation 38

D. Additional discussions 42

D1. Limitations of using reproduction number 42

D2. Using stringency index of NPIs 43

D3. Herd immunity threshold 44

Reference 45

A. Methodology

A1. Derivation of the instantaneous reproduction number from SIR model

In this study, the used instantaneous reproduction number, denoted as R_t, was estimated by Arroyo-Marioli et al.[1]. Here we give a brief description of how to derive R_t from the SIR model. The basic reproduction number, denoted R_0 , is an important epidemiological parameter, representing the expected number of infections caused by an infected person during the corresponding infectious period when everyone else is susceptible[2]. When $R_t < 1$, the average number of second-generation infections will be less than 1, where the epidemic will gradually vanish. Assuming there are no significant natural births and deaths, the standard SIR model without any intervention can be described as

$$dS = -\beta IS$$

$$dI = \beta IS - \gamma I$$

$$dR = \gamma I$$

where β is the transmission rate and γ is the recovery rate. Here, the disease-free equilibrium of the above epidemiological model can be used to derive R_0 from the regeneration matrix. Specifically, assuming

$$dx/dt = f(x) = r(x) - h(x)$$

where $r(x)$ is the ratio of new infection, $h(x)$ is the transfer rate that infections moved to the recovered population. The regeneration matrix is defined as

$$FV^{-1} = dr/dx * (dh/dx)^{-1}$$

Now, the basic reproduction number, R_0, is the spectral radius of the regeneration matrix, i.e., the eigenvalue with the maximum norm. With respect to the above standard SIR model, the regeneration matrix is

$$FV^{-1}=d(\beta IS)/dI * (d(\gamma I)/dI)^{-1} = \beta S/\gamma$$

and the disease-free equilibrium is (1,0,0). Thus, the basic reproduction number is

$$R_0 = \beta/\gamma$$

The instantaneous reproduction number, denoted R_t, is defined as

$$R_t = R_0 * (S/S + I + R)$$

which is equal to the average number of individuals infected by a single infection when a fraction $(S/S + I + R)$ of the population is susceptible. Finally, the daily growth rate (g_t) of the number of infections is

$$g_t = (I_t - I_{t-1})/I_{t-1} = \gamma(R_t - 1)$$

The adoption of an SIR model, rather than a SEIR model, in the method of Arroyo-Marioli et al.[1] was based on some considerations below. To use the SEIR model, they would have to estimate the number of currently exposed individuals. Doing so would triple the number of model parameters, such as average duration of the incubation period, relative infectiousness of exposed and infectious individuals. In simulations, they found that their estimator derived from the SIR model produces accurate estimates even when the true model is SEIR rather than SIR.

A2. Calculation of the instantaneous basic reproduction number

The basic reproduction number is affected by several factors, including the duration of infectivity of infected people, the infectiousness of the microorganism, and the number of susceptible people in the population in contact with infected people. Within the same local context, because of the variation in infectiousness between different variants of SARS-CoV-2, different COVID-19 variants are assumed to have different estimates of R_0. We estimated the instantaneous basic reproduction number, denoted $R_{0,t}$, for each country in order to account for different variants associated with COVID-19 cases over

time. We used the weighted average of the basic reproduction number of each SARS-CoV-2 variant as the instantaneous basic reproduction number,

$$R_{0,t} = \sum_{i=1}^{7} w_{i,t} R_{0,i}$$

where $w_{i,t}$ is the weight of the basic reproduction number of the coronavirus i, $R_{0,i}$, at day t, calculated by the proportion of infections caused by that virus. Noting that the transmissibility of variants was mainly reported by the expansion of the basic reproduction number of SARS-CoV-2 strains before variants of concern (VOCs) predominantly transmitted in communities[3,4,5]. We assumed that the coefficient of expansion followed a normal distribution where the parameter of variation was determined by the consequent 95% credible interval listed in Supplementary Table 1. Additionally, despite the existence of policy fatigue[6], population behaviour amid our study period might have been altered by long-term NPI implementation compared to before the COVID-19. Therefore, we used the highest R_t between 1 August 2020 to 1 December 2020 before vaccines rolled out, as the prior mean over R_0 of SARS-CoV-2 for each country.

$$R_0 \sim \text{gamma}(max_{2020-08-01<t<2020-12-01} R_t, k),$$

$$k \sim \text{Half normal}(0, 0.5),$$

reflecting the potential COVID-19 transmissibility in the WHO European Region during the period of NPI relaxation after the first wave (Supplementary Fig. 1).

Overall, compared with the estimates of R_0 of SARS-CoV-2 in the first wave[7], the transmissibility in the second half of 2020 has been weakened (Supplementary Table 2). Despite the reduced adherence to NPIs, the instantaneous basic reproduction number of Covid-19, denoted as $R_{0,t}$, decreased to a median of 1.8 (IQR: 1.7 - 2.3) (Fig. 2) from an average R_0 for the first wave in Western Europe of 2.2 (95% CI: 1.9 - 2.6)[8].

However, $R_{0,t}$ increased as new variants emerged over time. We found that $R_{0,t}$ increased to 2.1 (IQR: 1.9 - 2.7) when the Alpha variant became the dominant strain of COVID-19 in circulation in February 2021, and increased further to 2.6 (IQR: 2.1 - 3.3) in June 2021 when the Delta variant became the dominant strain of infection.

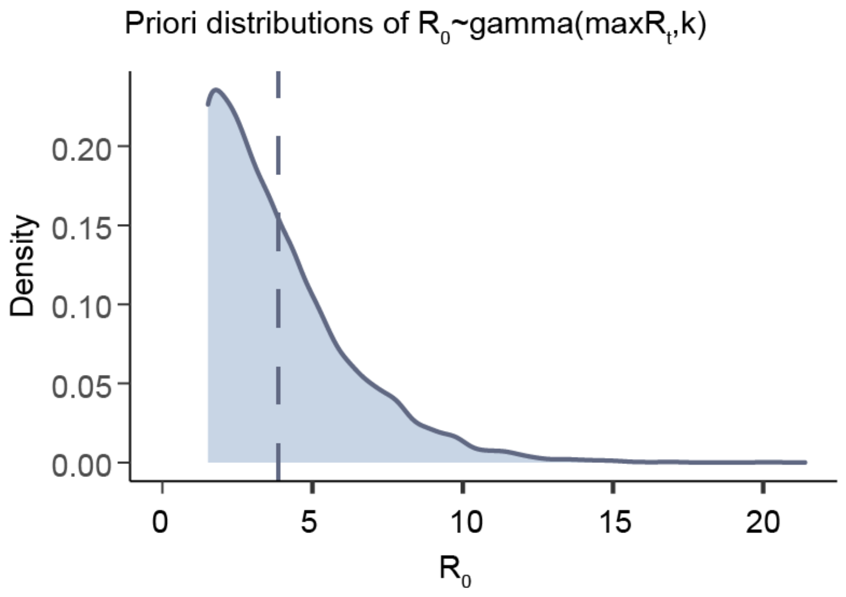

Supplementary Fig. 1 The prior distribution of the basic reproduction number (R_0) of SARS-CoV-2 in initial outbreaks in the United Kingdom.

Supplementary Table 1. Transmissibility of SARS-CoV-2 variants. The estimates are shown by its medians and IQR. These parameters were used in the calculation of synthetic basic reproduction numbers.

**Variants**	**Coefficient of expansion regarding R_0 of SARS-CoV-2 (Q1-Q3)**	**Source**
Alpha	1.29 (1.24-1.33)	Curran, J., et al.[3]
Beta	1.25 (1.20-1.30)	CDC[4]
Gamma	1.38 (1.29-1.48)	Campbell, et al.[5]
Delta	1.97 (1.76-2.17)	
Eta	1.29 (1.23-1.35)	
Kappa	1.48 (1.28-1.69)	

Supplementary Table 2. The basic reproduction number of SARS-CoV-2 across countries.

Country	Highest R_t*	First wave R_0**	Country	Highest R_t*	First wave R_0**
Austria	1.6	2.88	Iceland	2.12	-
Belgium	1.58	2.87	Israel	1.35	3.19
Bulgaria	1.63	2.66	Italy	1.71	2.77
Switzerland	2	2.42	Liechtenstein	1.91	-
Cyprus	1.96	-	Lithuania	1.7	2.19
Czechia	1.64	2.69	Luxembourg	1.76	3.23
Germany	1.49	2.35	Latvia	1.81	2.26
Denmark	1.69	1.74	Netherlands	1.66	2.05
Spain	1.57	3.3	Norway	1.7	2.05
Estonia	1.89	1.86	Poland	1.73	2.36
Finland	1.38	2.48	Portugal	1.46	2.5
France	1.48	2.64	Slovakia	1.57	2.77
United Kingdom	2.05	2.99	Slovenia	1.72	1.78
Croatia	1.75	2.54	Sweden	1.81	1.82
Hungary	2.34	2.8	Ukraine	1.24	4.6
Ireland	1.62	2.09			

*Highest R_t: the highest R_t estimated by Arroyo-Marioli et al.[1] between 1 August 2020 to 1 December 2020.
**First wave R0: the estimates[7] of the basic reproduction number in the first wave of COVID-19, the absent country-specific estimates are set as blank.

A3. Calculation of the practical vaccination rate

The reported fully vaccination rates across countries were the proportion of the whole population who have received all doses prescribed by the vaccination protocol. While the de facto population that is immune is lower than the fully vaccinated population since vaccines are not 100% effective to prevent COVID-19 infection among individuals. It is an important indicator of the immune population in the estimation of the effect of vaccination. For example, assuming that the basic reproduction number of Delta is approximately 5, it is estimated that an immune population proportion of 80 percent is required in order to prevent an increasing spread of COVID-19. Under these circumstances, even if 100 percent of national populations are vaccinated, an efficacy

of vaccines against the Delta variant is still required being 80% in order to halt transmission. As different COVID-19 vaccine products with various efficacy have been used across countries, to integrate vaccination data from different vaccines and countries for modelling their overall effect, we termed the fraction of the de facto population with immunity as a result of vaccination as the practical vaccination rate.

Here, we evaluated the biweekly practical vaccination rate (V_t) according to the used vaccine products across the 31 study countries. We estimated the fraction of the population protected by the specific vaccine product i by multiplying the documented efficacy of vaccine product i (e_i) against SARS-CoV-2 before VOCs became predominant, with the proportion of the vaccine product i used ($p_{i,t}$). The fraction of the population with immunity attributed to each vaccine product i was then evaluated by summing this number for all the six vaccines involved in this study. The practical vaccination rate was estimated by the reported fully vaccination rate multiplying the former sum. That is,

$$V_t = fully\ vaccination\ rate_t \sum_{i=1}^{6} e_i * p_{i,t}$$

The efficacy of six vaccines against SARS-CoV-2 is listed in Supplementary Table 3.

Supplementary Table 3. Efficacy of various vaccines against SARS-CoV-2 in clinical trials. The estimates are shown by its medians and IQR.

Vaccine product	Efficacy	Vaccine product	Efficacy
Sinopharm.Beijing[9]	78.10 (IQR: 64.90,86.30)	AstraZeneca[12]	66.70 (IQR: 57.40,74.00)
Sputnik.V[10]	91.60 (IQR: 85.60,95.20)	Moderna[13]	94.10 (IQR: 89.30,96.80)
Pfizer[11]	95.00 (IQR: 90.30,97.60)	Johnson & Johnson[14]	66.90 (IQR: 59.10,73.40)

A4. Index of openness risk

In April 2020, the WHO proposed six domains of measures that governments needed to deploy to diminish the risks of easing NPIs[15]. Here we give a summary of the six domains and how OxCGRT calculated the index of openness risk at day t based on them.

1. COVID-19 transmission is controlled to a level that could be handled by health care capacity.

$$CC = \Delta cases_t/50$$

   where $\Delta cases_t$ is the average new daily cases from the last 7 days. Cases controlled is defined as 1 if $\Delta cases_t >= 50$.

2. Sufficient public health workforce and health system capacities are in place.

$$TT = 0.25(1 - \frac{H2}{3}) + 0.25(1 - \frac{H3}{2}) + \frac{0.5\left(\ln\left(tests_{global\_max}\right) - \ln(tests)\right)}{\ln\left(tests_{global\_max}\right) - \ln\left(tests_{global\_min}\right)}$$

   where H2 is the value of the testing policy indicator (H2) in OxCGRT database, H3 is the value of the contract tracing policy indicator (H3) in OxCGRT database, $\ln(tests)$ is the natural logarithm of the number of tests-per-case conducted by the underlying country, and $\ln(tests_{global\_min/max})$ is the natural logarithm of the number of tests-per-case conducted by the country that has the most/least tests-per-case.

3. Outbreak risks in high-vulnerability settings are minimised. This category has not been considered in the calculation.

4. Preventive measures are established in workplaces. This category has not been considered in the calculation.

5. Risk of exporting and importing cases from communities with high risks of transmission is managed.

$$M = \begin{cases} = 1 & if\ C8 = 0 \\ = 0.5 & if\ C8 = 1 \\ = 0.25 & if\ C8 = 2 \\ = 0 & if\ C8 = 3{,}4 \end{cases}$$

where C8 is the value of the international restrictions policy indicator in the OxCGRT database.

6. Communities are fully engaged.

$$C = 0.5CC + \frac{(1 - 0.5CC)(mob - 20)}{100}$$

where mob is the level of mobility as a percentage of pre-COVID baseline levels reported by Apple (average of all three reported mobility types) or Google (average of "retail and recreation", "transit stations", and "workplaces" mobility types). Of note, if the value of public information campaigns (H1) in OxCGRT is not equal to 2, the metric is set to 0 as there is no national public information campaign. The mob is set as the highest value generated by Apple and Google. If neither Apple nor Gooogle mobility data is available, the metric is left blank. mob is set to 20 if it is less than 20 and set to 120 if it is higher than 120.

OxCGRT collected information relevant to domain 1, 2, 5, and 6, summarised in Supplementary Table 4. The risk of openness index can be directly calculated by the average of the above four metrics as:

$$RoOI_{unadjusted} = Mean(CC, TT, M, C)$$

Instead of adjusting the risk of openness index by the cases controlled by the country itself. We used the vaccination rate as a measure of closeness to herd immunity. Then, the adjusted risk of openness index can be calculated as:

$$RoOI_{adjusted} = (1 - vaccination) + vaccination * RoOI_{unadjusted}$$

Ideally, when everyone has received all doses prescribed by the vaccination protocol, the risk of removing public health measures will become $RoOI_{unadjusted}$, which is a case-evidenced openness risk.

Supplementary Table 4. Summary of sub-indices used to calculate the index of openness risk

WHO categories	Data sources	Risk index sub-component
Transmission controlled	Daily cases and deaths data from European Centre for Disease Prevention and Control[16] and John Hopkins University CSSE COVID-19 Data Repository[17]	A metric between 0 and 1 based on new cases confirmed each day. This is captured in two dimensions: 1. A measure to account for a localised outbreak. 2. An 'endemic factor' that modulates the index according to the total number of new cases
Test / trace / isolate	● OxCGRT: H2 (testing policy)<br>● OxCGRT: H3 (contact tracing policy)<br>● Testing data from Our World in Data[18]	A metric between 0 and 1; half based on testing and contact tracing policy, and half based on the number of tests-per-case a country has conducted. (does not measure isolation)
High vulnerability settings	-	-
Preventative measures established in workplaces	-	-
Manage risk of exporting and importing cases	OxCGRT: C8 (international travel restrictions)	A metric between 0 and 1 based on the stringency of the country's restrictions on travel arrivals. (does not measure risk of exporting cases)
Communities understanding and behaviour change	● OxCGRT: H1 (public information campaigns)<br>● Travel and mobility data from Apple[19] and Google[20].<br>● Daily cases and deaths (from European CDC via Our World in Data)	A metric between 0 and 1 based on whether a country has a public information campaign and the level of mobility reduction, weighted for current transmission risk.

B. Additional results

B1. Effect of temperature and unknown factors

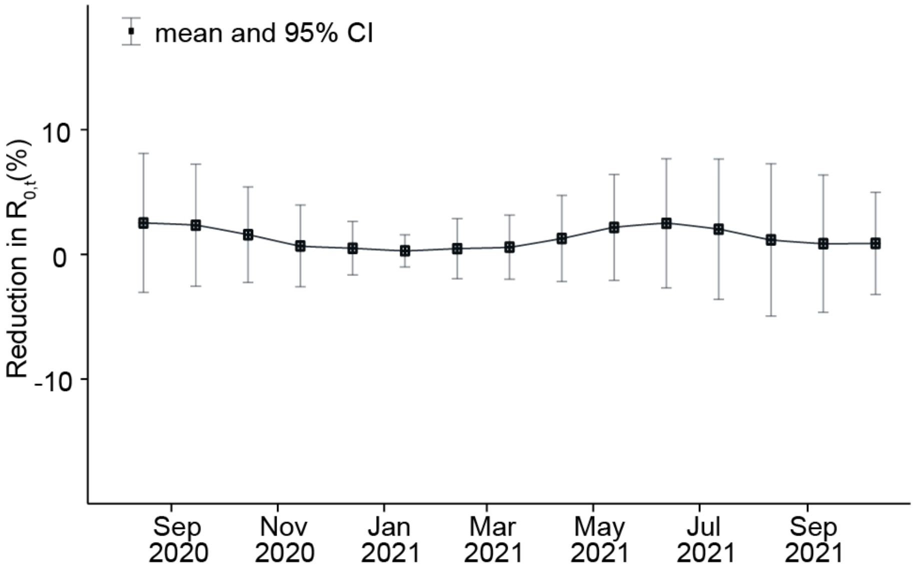

Supplementary Fig. 2 The effect of air temperature over time.

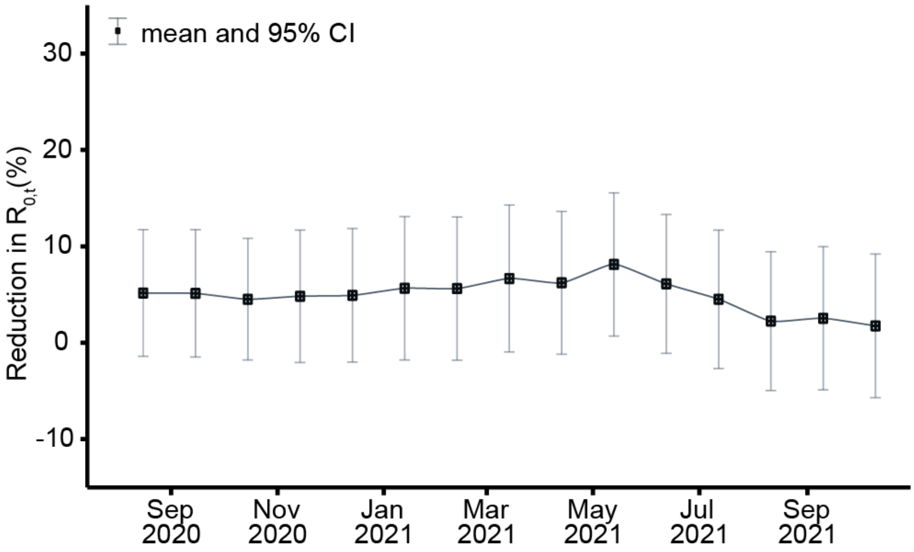

Supplementary Fig. 3 The effect of unknown factors over time.

B2. Country-specific effect NPIs and vaccination over time

Due to the heterogeneous context across the 31 countries, we estimated the national effect of NPIs and vaccination for each of the 31 countries (Supplementary Fig. 4). Then, we used a meta-analysis of the data to generate the general effects of NPIs and vaccination, respectively, using a bottom-up approach.

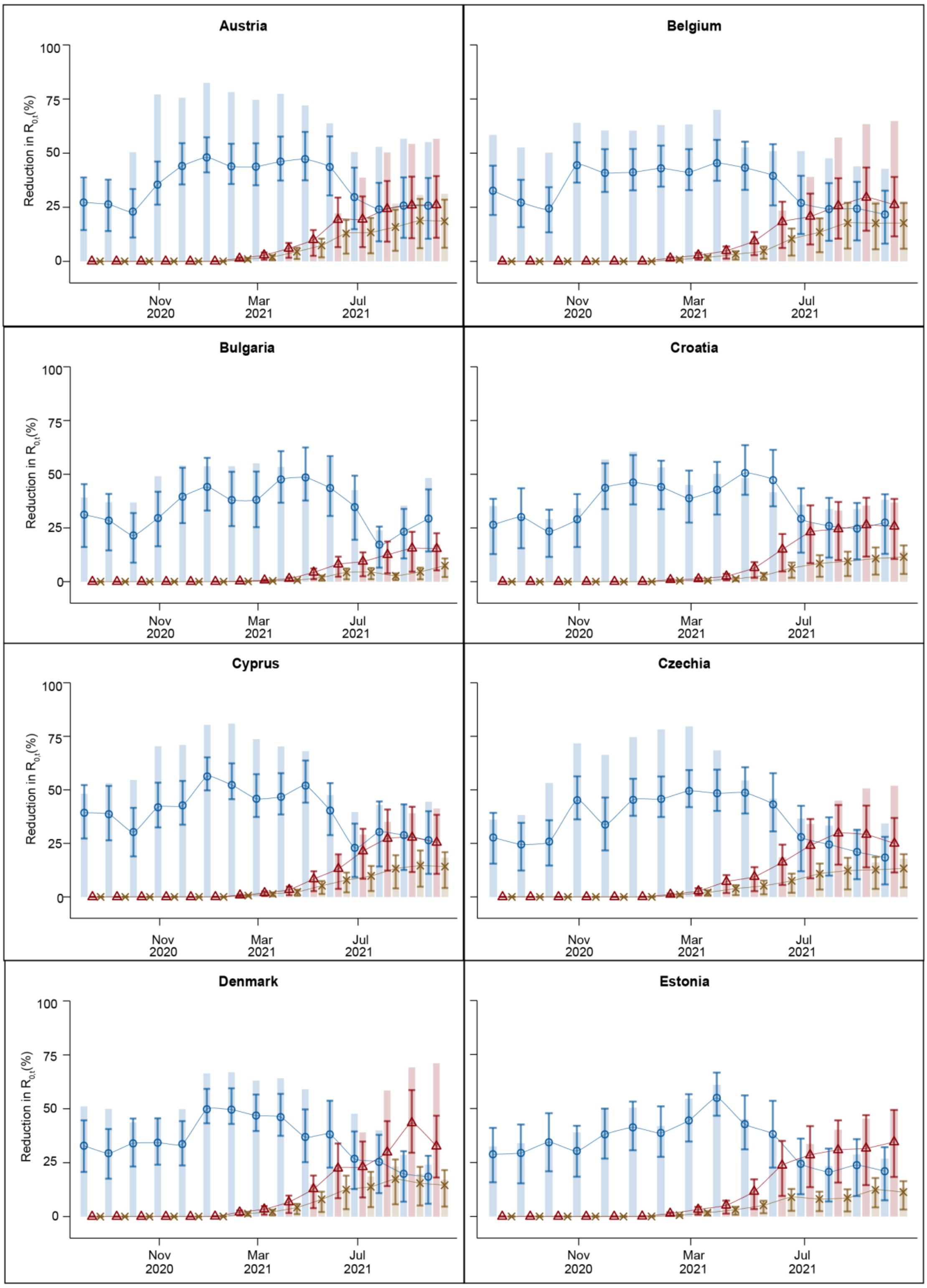

Supplementary Fig. 4 The effect of NPIs and vaccination for each of 31 study countries over time. The effects of NPIs (blue lines), vaccination (red lines) and interaction of SI and vaccination (yellow lines) were estimated for each month and country. The corresponding stringency index of NPI implementation and the practical vaccination rate over time were represented by the height of bars in the background.

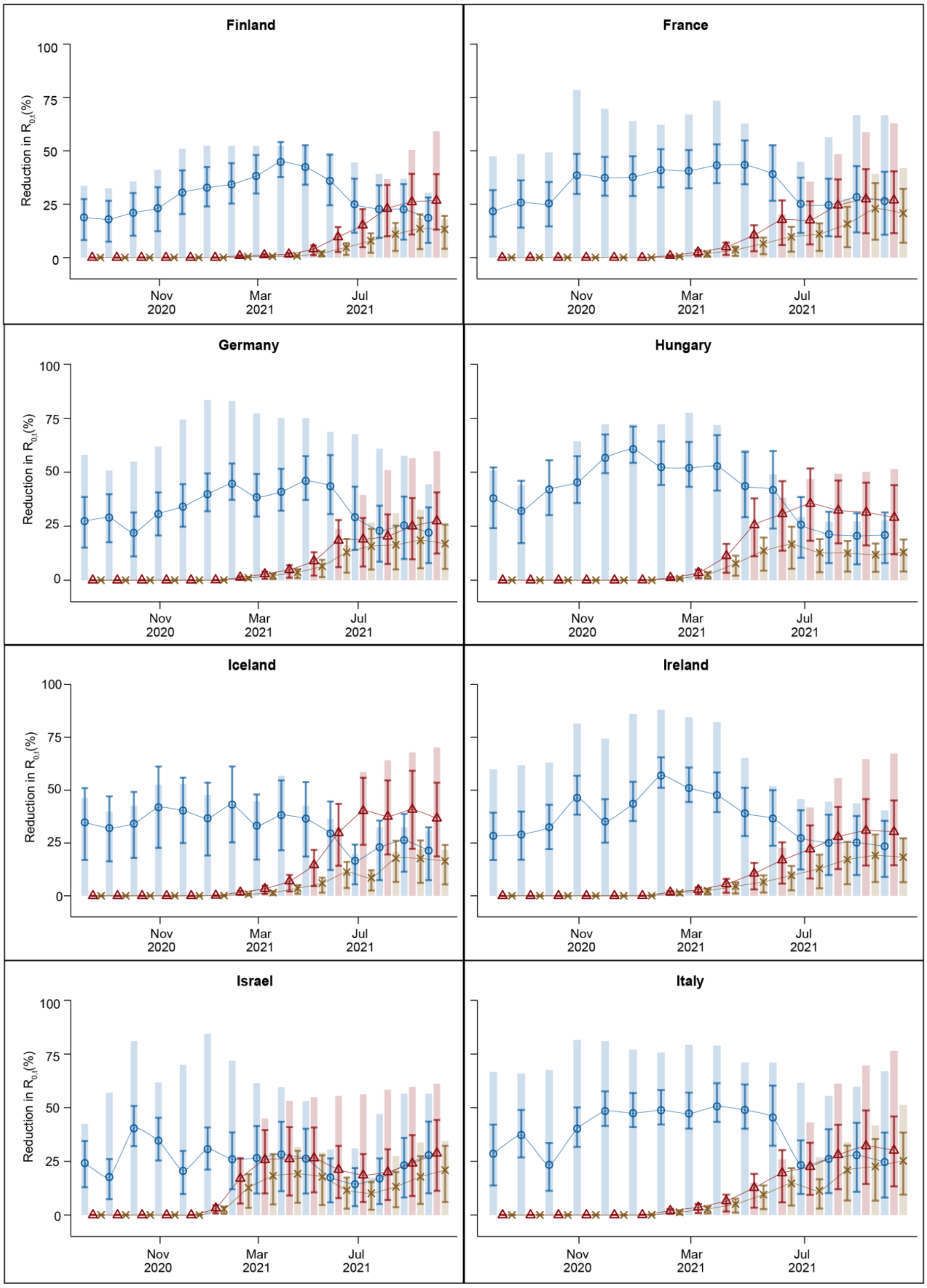

Supplementary Fig. 4 (Continue).

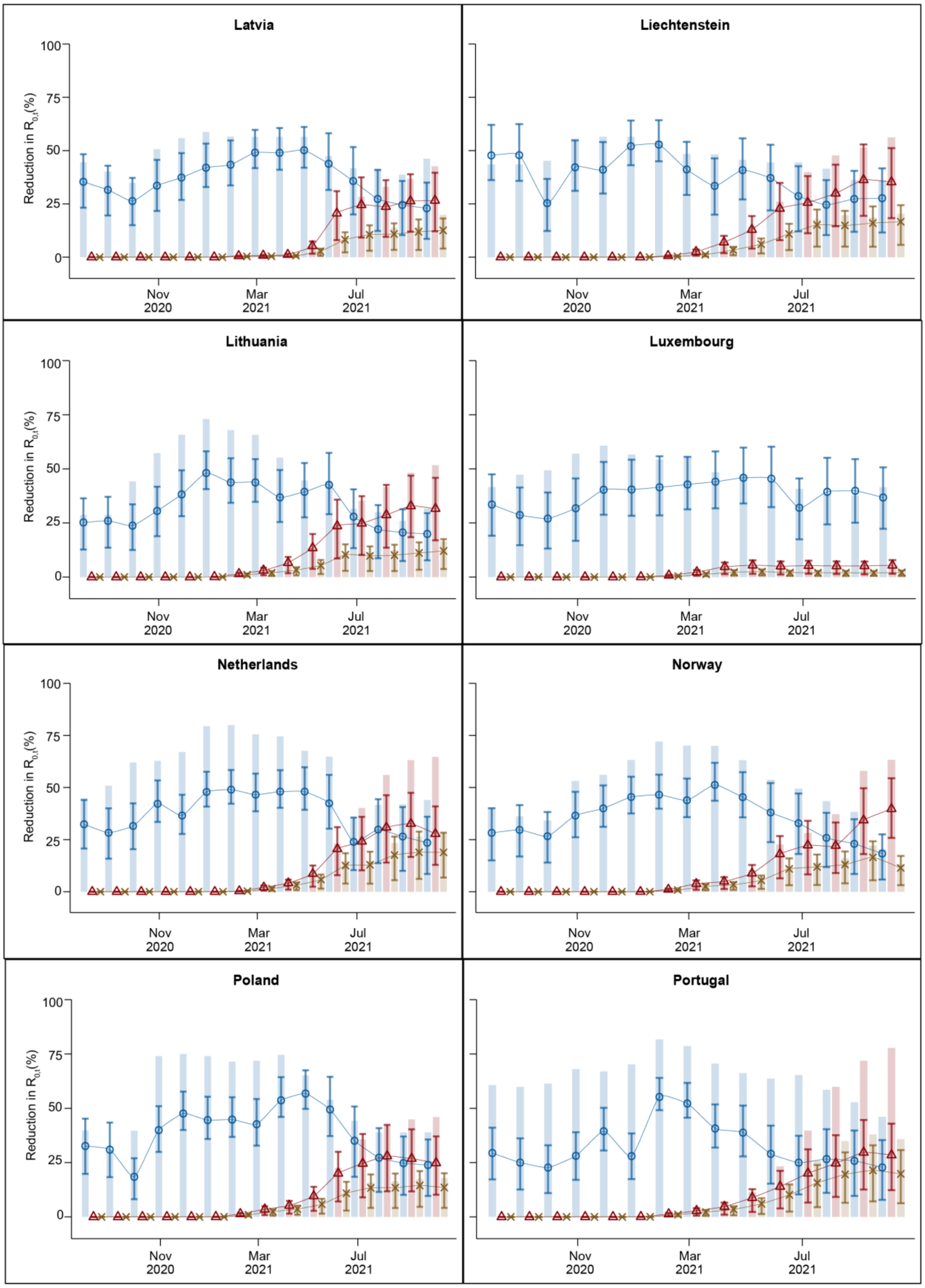

Supplementary Fig. 4 (Continue).

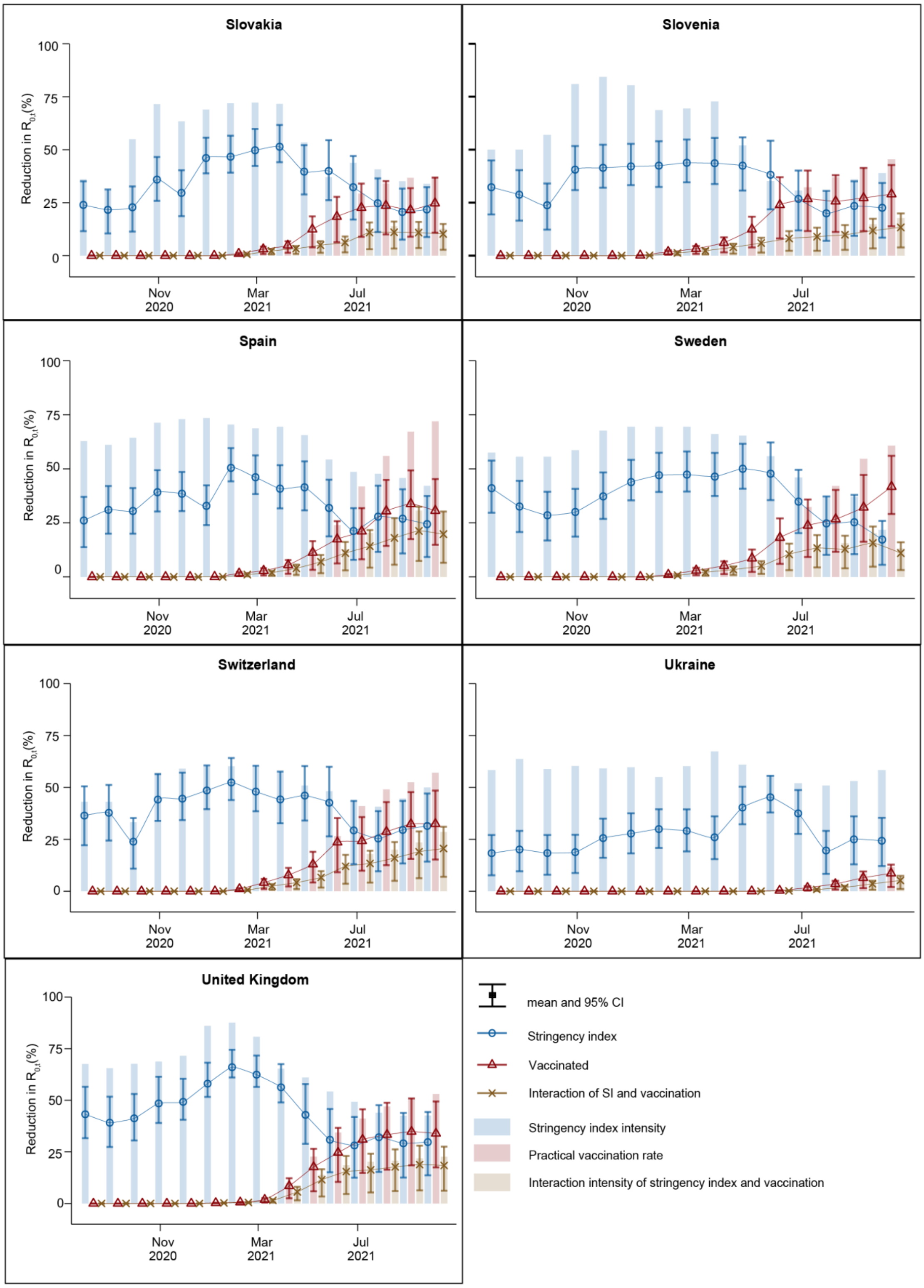

Supplementary Fig. 4 (Continue).

Instead of fitting separate Bayesian inference models for the study 31 countries, we also fit a full Bayesian model with fixed pooling, i.e., we run one inference over the whole dataset. The results in Supplementary Fig. 5 showed that the separate models are consistent with the full model based on our model assumptions.

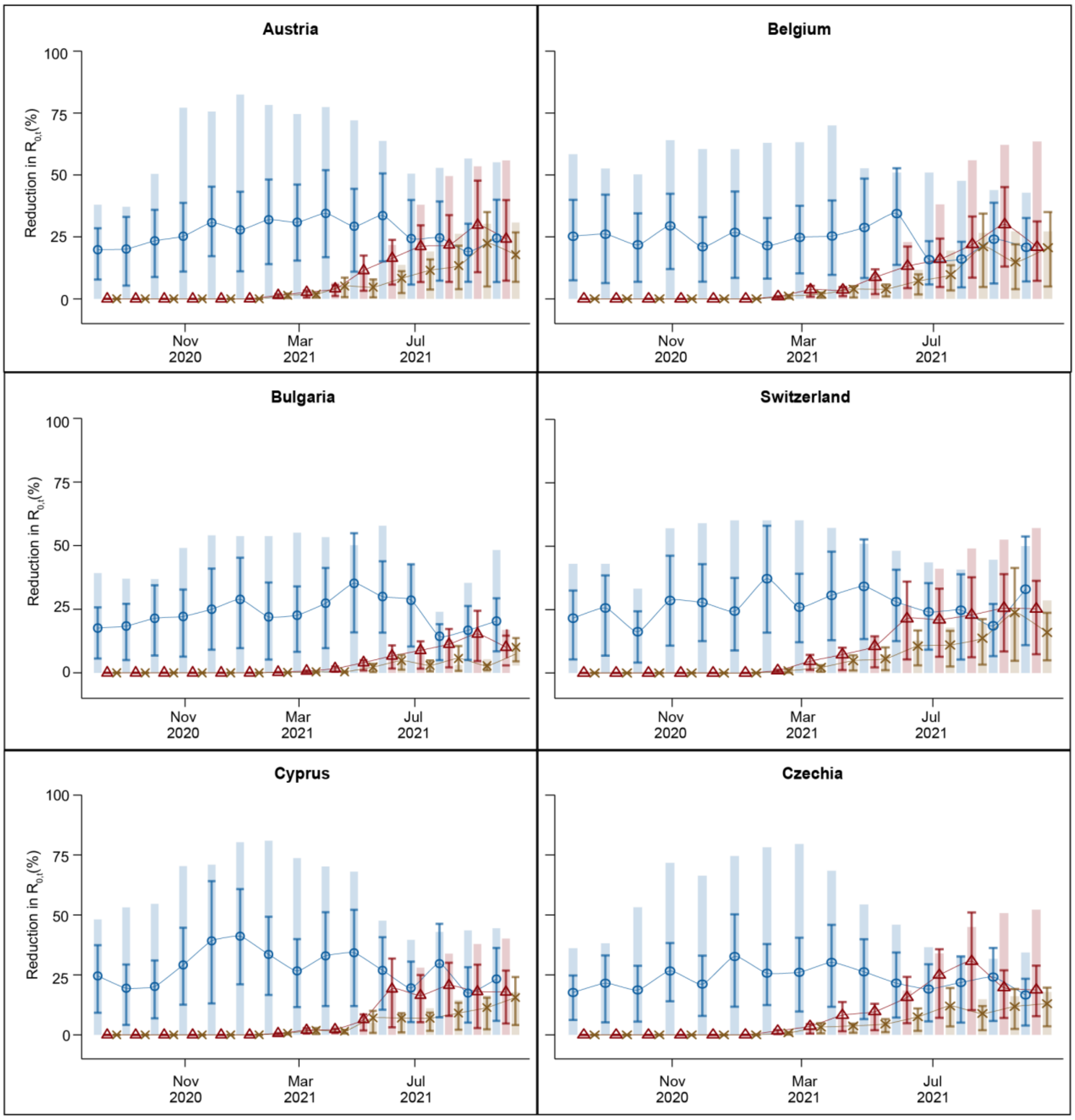

Supplementary Fig. 5 The effect of NPIs and vaccination for each of 31 study countries over time within the pooling model. The effects of NPIs (blue lines), vaccination (red lines) and interaction of SI and vaccination (yellow lines) were estimated for each month and country. The corresponding stringency index of NPI implementation and the practical vaccination rate over time were represented by the height of bars in the background.

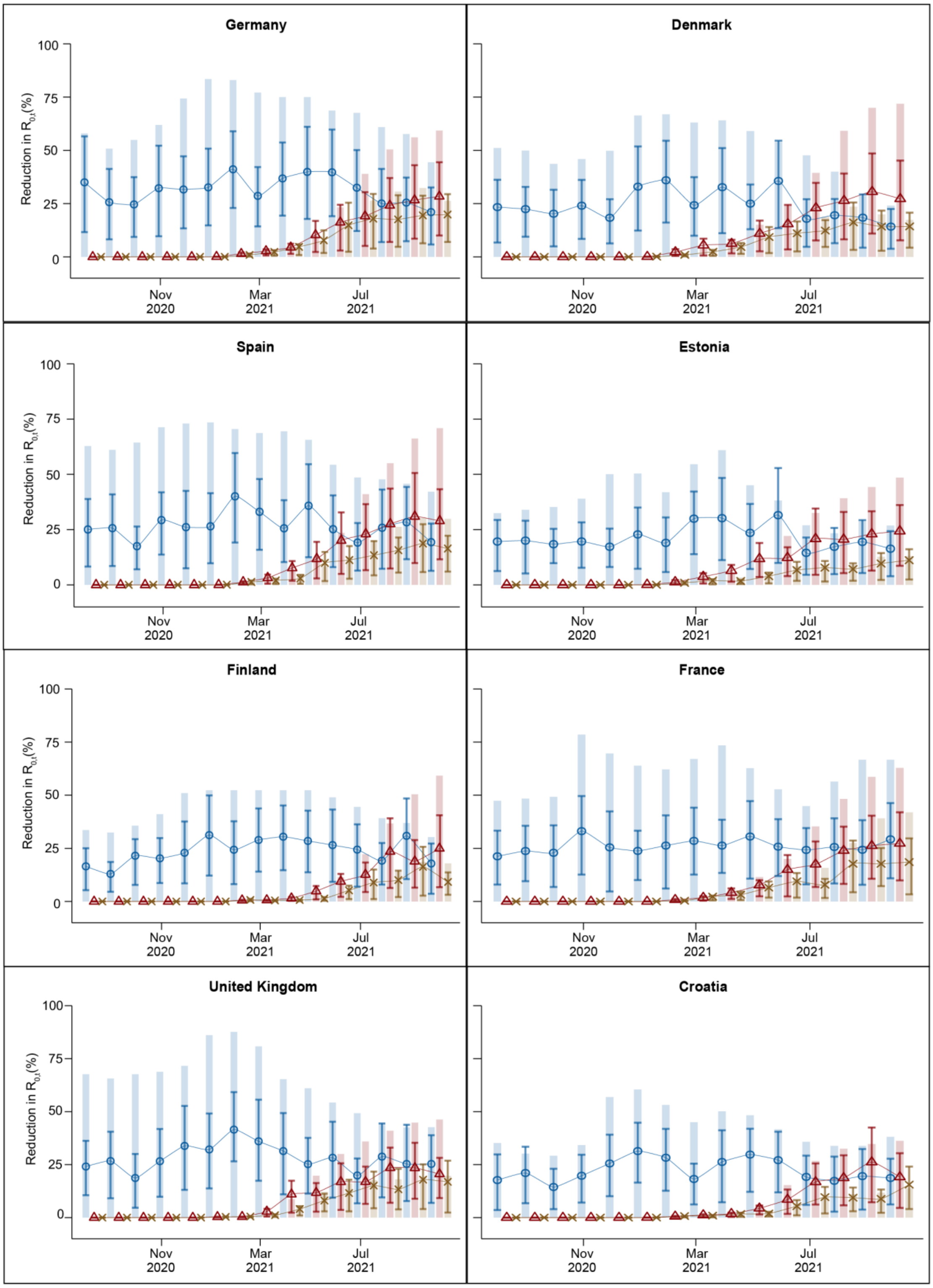

Supplementary Fig. 5 (Continue).

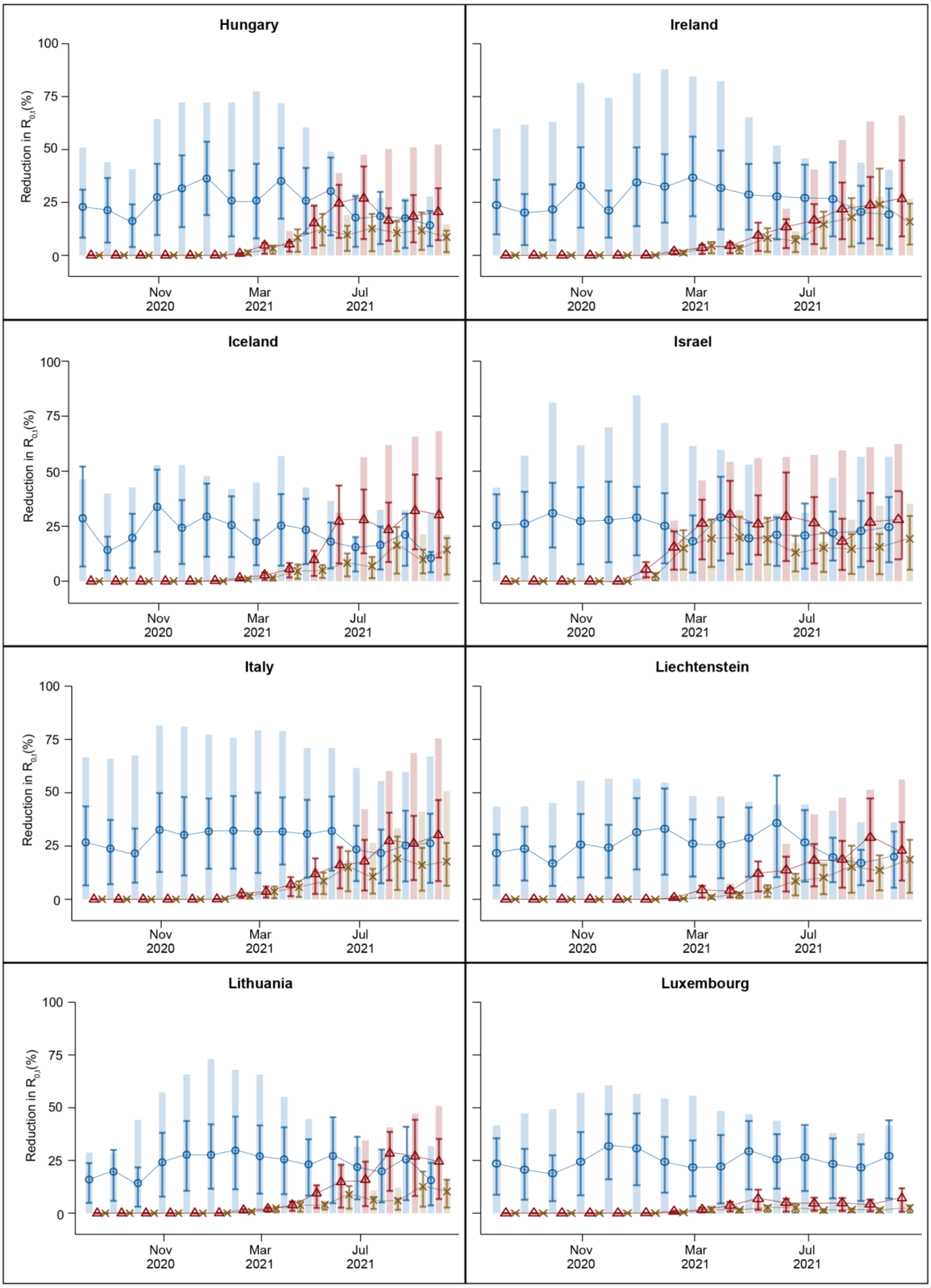

Supplementary Fig. 5 (Continue).

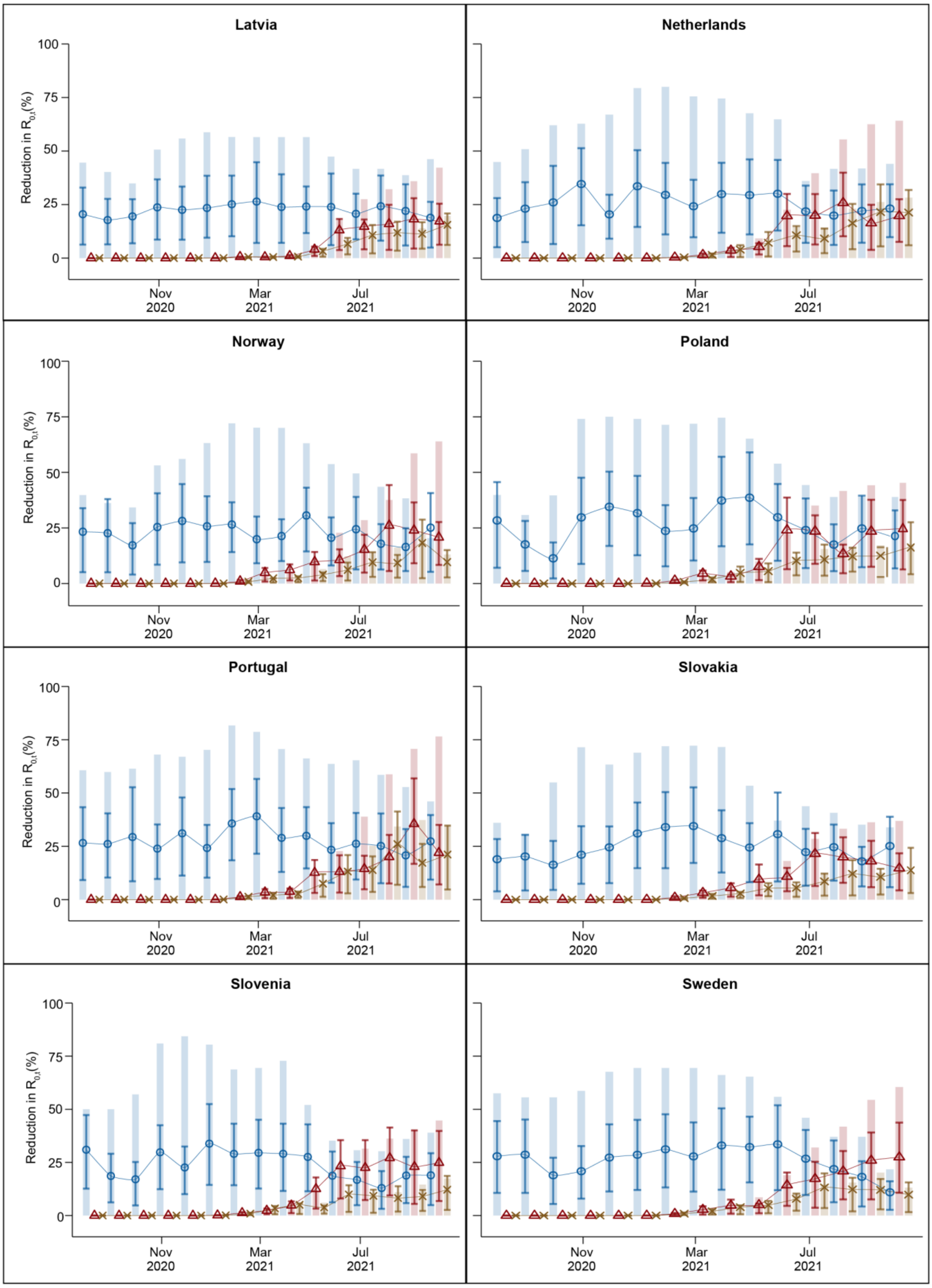

Supplementary Fig. 5 (Continue).

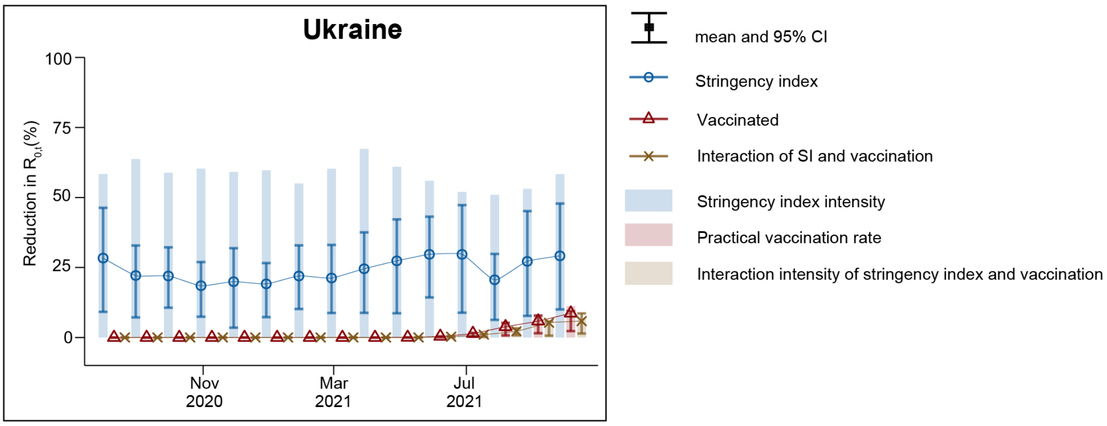

Supplementary Fig. 5 (Continue).

B3. Estimates of the coefficients

To estimate the effect of NPIs and in the presence of vaccination, we built a Bayesian model as follow,

$$R_t^c = R_{0,t}^c \exp\left(-\alpha_l^c N_t^c - \beta_l^c V_t^c - \lambda_l^c N_t^c V_t^c - \varphi_l^c T_t^c - \Delta_l^c\right) = \Phi_{t,l}^c$$

where N_t^c, V_t^c, and T_t^c are the stringency index of NPIs, practical vaccination rate, and air temperature for country c in month l at day t, respectively. In addition to NPIs and vaccination, we also modelled their interaction of reducing R_t by directly incorporating a product term $(N_t^c V_t^c)$ in our model. Moreover, the unobserved confounders of the change between $R_{0,t}^c$ and R_t^c were represented by the residual Δ_l^c. To estimate the model parameters, we used a Bayesian framework to provide the estimates with prior knowledge. We assumed that $R_t^c \sim \text{gamma}\left(\Phi_{t,l}^c, 0.5\right)$. As NPIs and vaccination were likely to positively impact the trajectories of COVID-19, i.e., reducing $R_{0,t}$, we put a gamma prior with hyperprior over the coefficients of NPIs, vaccination and their interaction term in our model. Specifically, α_l^c, β_l^c and λ_l^c, following $\text{gamma}(u, 1)$ and $u \sim \text{uniform}(0,1)$, varied by country according to their data contexts. Additionally, we had a Gaussian prior over the coefficients $\varphi_l^c \sim N(0, k)$ and $\Delta_l^c \sim N(0, k)$,

k~Half normal $(0, 0.3)$, as air temperature and the unseen factors were believed can either increase or decrease R_t. Supplementary Fig. 6 showed the prior distributions over the coefficients of our model. Supplementary Figs. 7 – 11 showed the estimates of coefficients in our model over time across 31 countries.

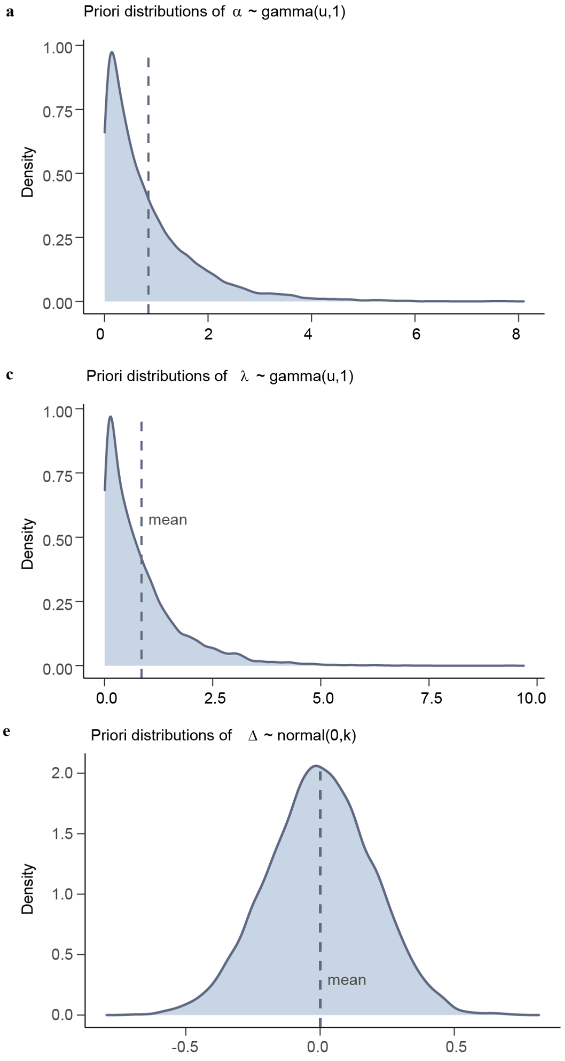

Supplementary Fig. 6 The prior distribution of coefficients over NPIs (a), vaccination (b), and their interaction (c), air temperature (d), and unseen factors (e).

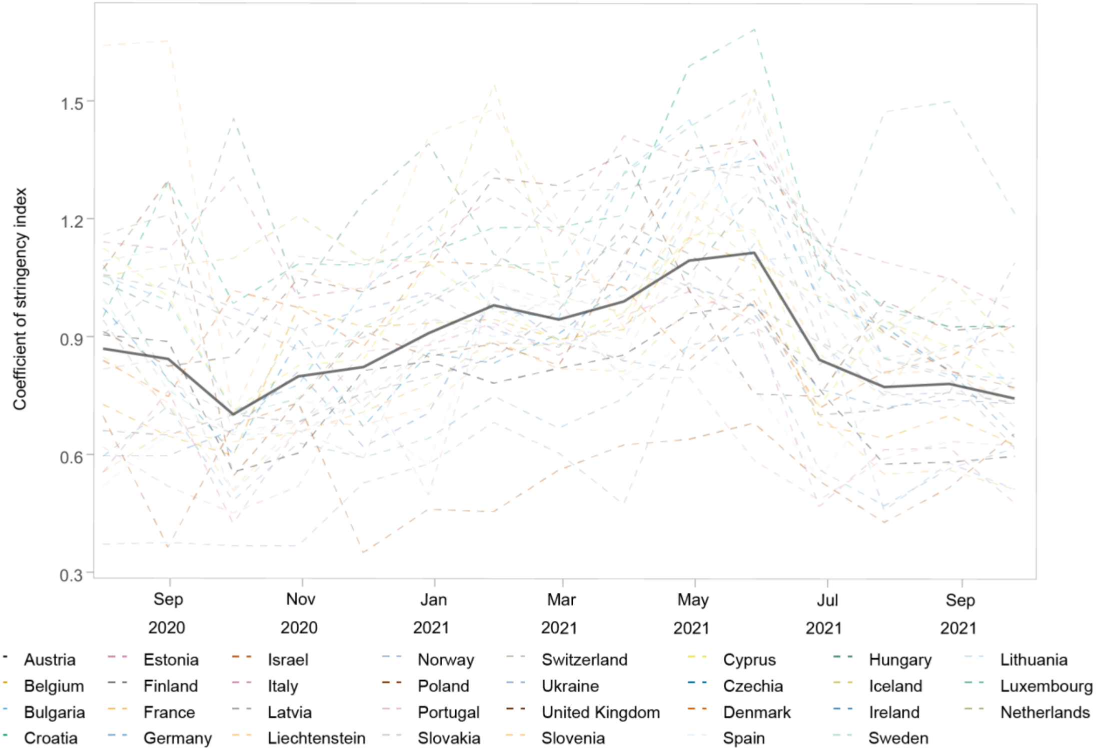

Supplementary Fig. 7 The estimates of coefficients for stringency index across countries and months.

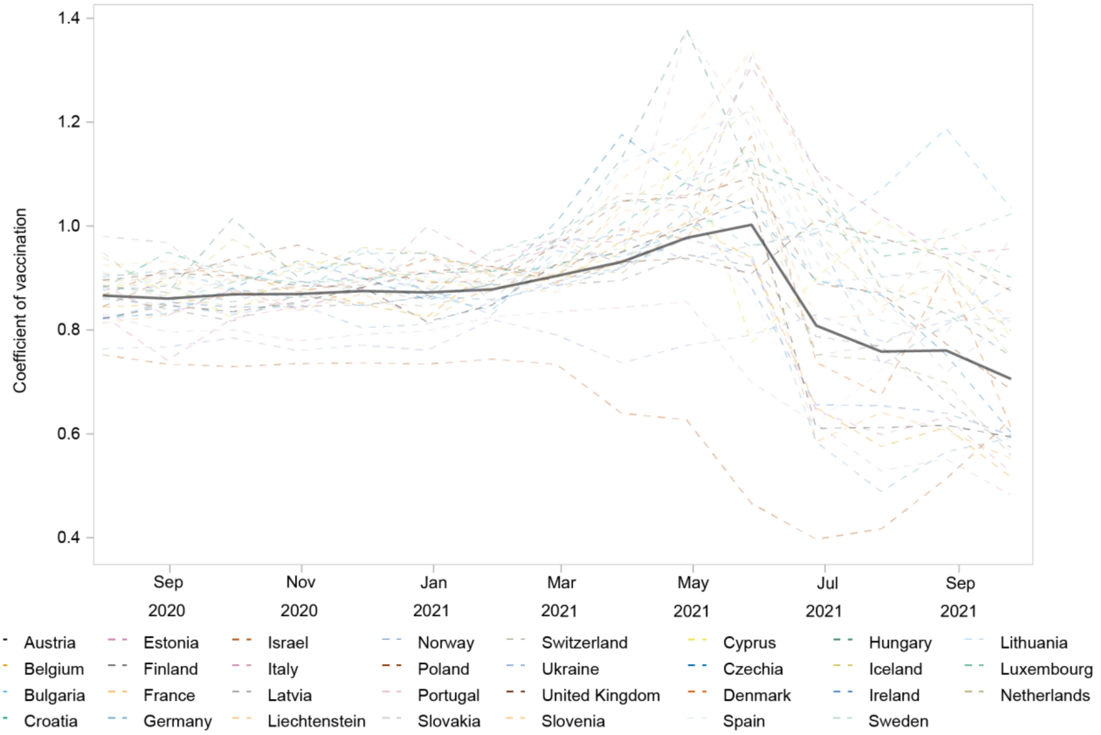

Supplementary Fig. 8 The estimates of coefficients for practical vaccination rate across countries and months.

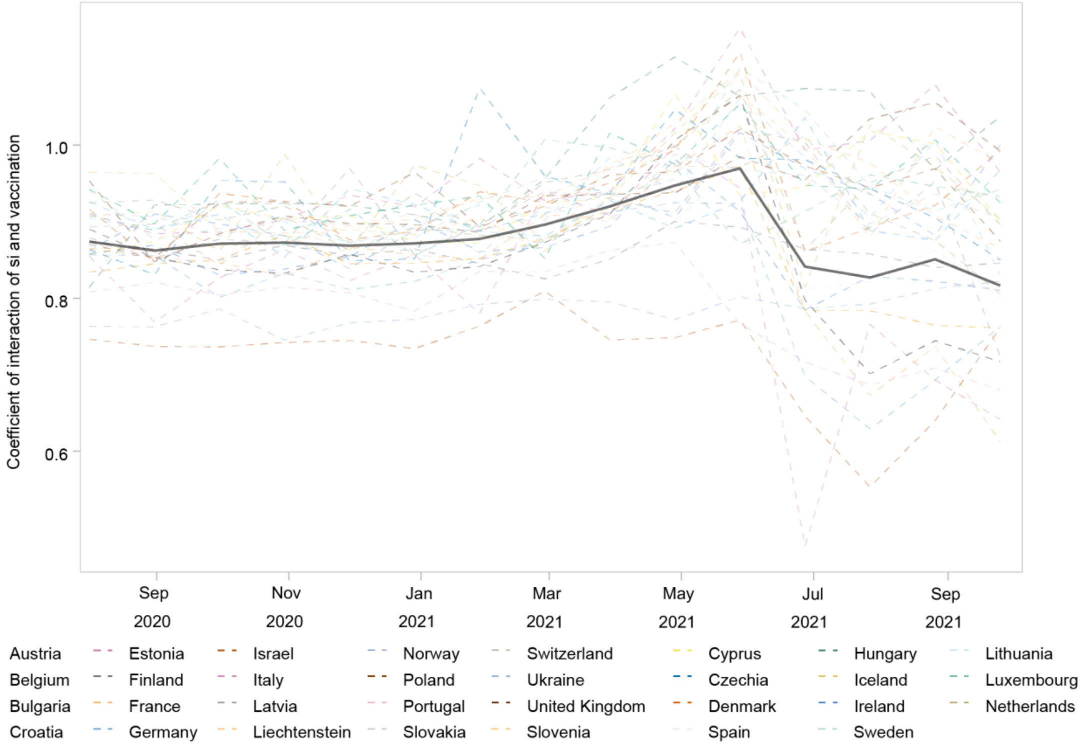

Supplementary Fig. 9 The estimates of coefficients for interaction of NPIs and vaccination across countries and months.

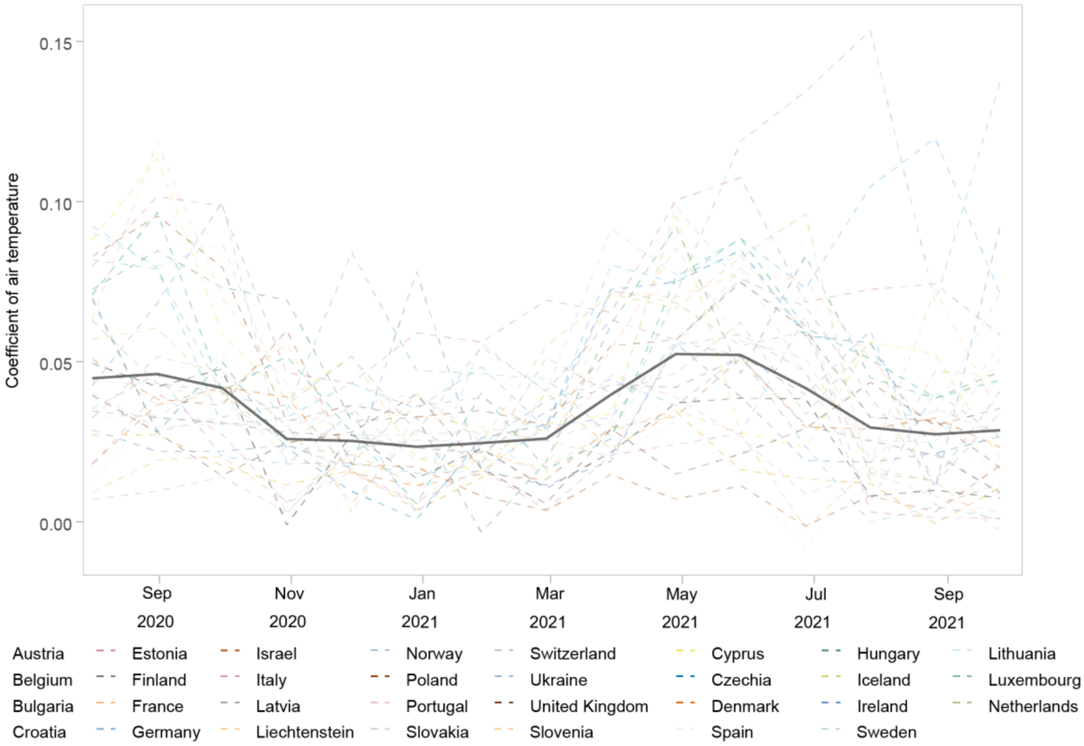

Supplementary Fig. 10 The estimates of coefficients for air temperature across countries and months.

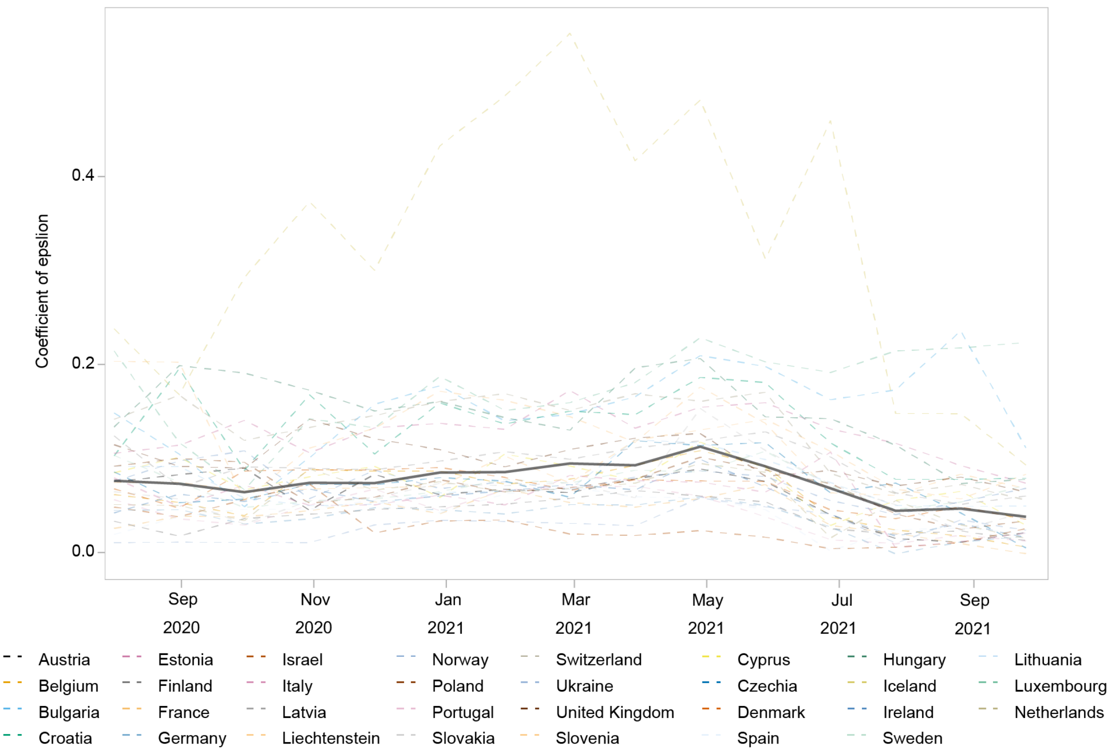

Supplementary Fig. 11 The estimates of coefficients for unobserved confounders across countries and months.

B4. Collinearity

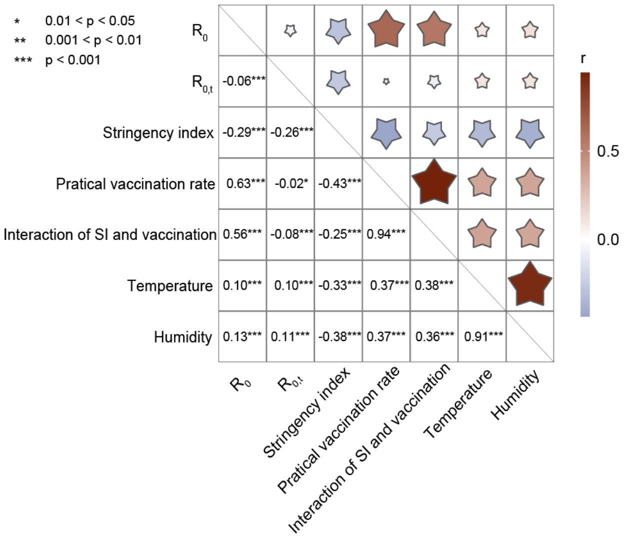

Supplementary Fig. 12 The collinearity between the involved variables in this study. The correlation coefficients were calculated by two sided Pearson method. Noting that the temperature is highly associated with humidity, we only take temperature as the control variable to account for the seasonal and calendar effect.

B5. Sensitivity analysis

We changed our settings of model structure and parameters selection in order to assess the robustness of our model. Specifically, we designed following scenarios:

S1. The prior mean over R_0 was further set as the estimates of R_0 in the first wave;

S2. The prior mean over R_0 was further set as the average of the highest R_0 and the estimates of R_0 in the first wave;

S3. The prior distribution of R_t was set as normal distribution;

S4. The prior distribution of R_t was set as Weibull distribution;

a

b

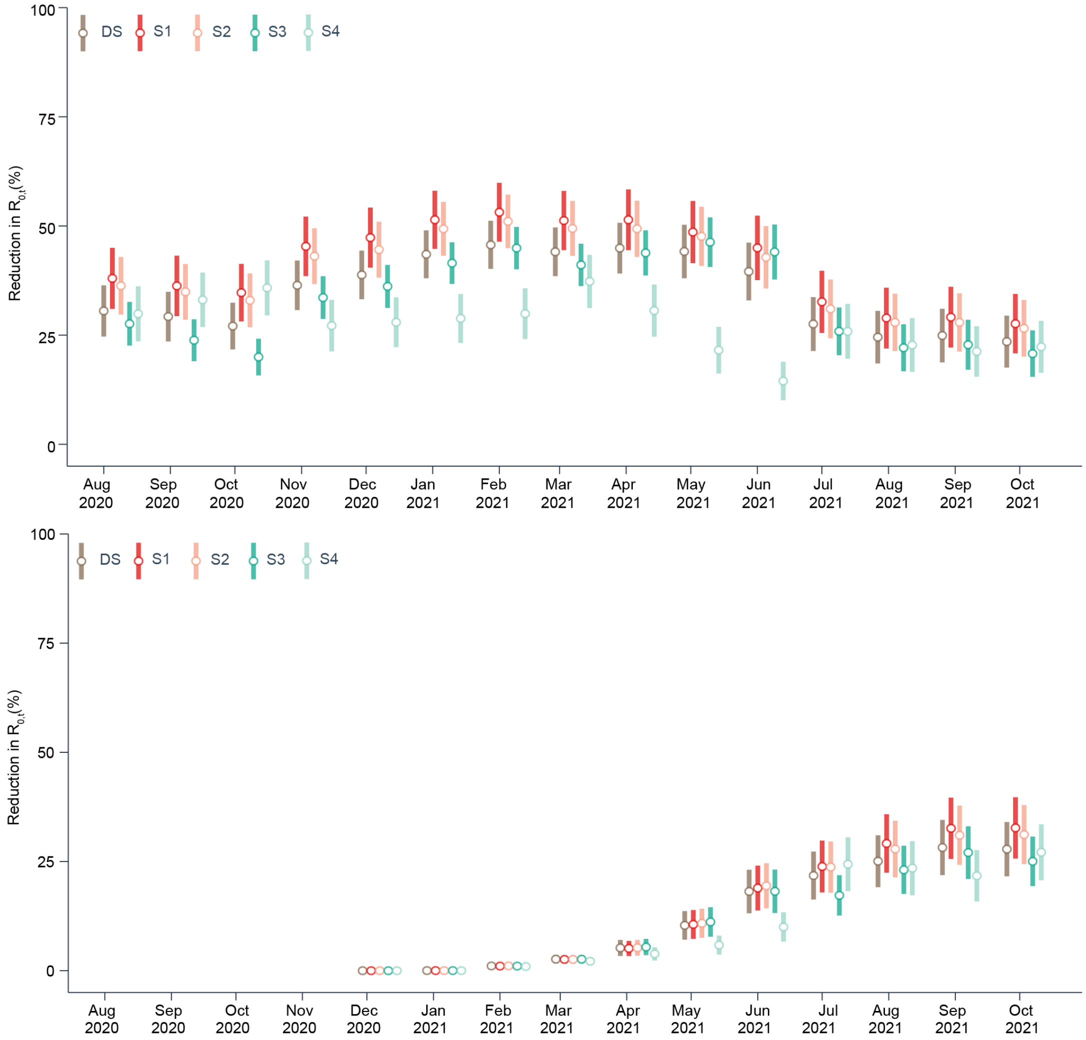

Supplementary Fig. 13 Effects of (a) NPIs, (b) vaccination, (c) interaction of NPIs and vaccination, (d) air temperature, and (e) unknown factors on reducing COVID-19 transmission over time under different model settings. DS is our default model setting.

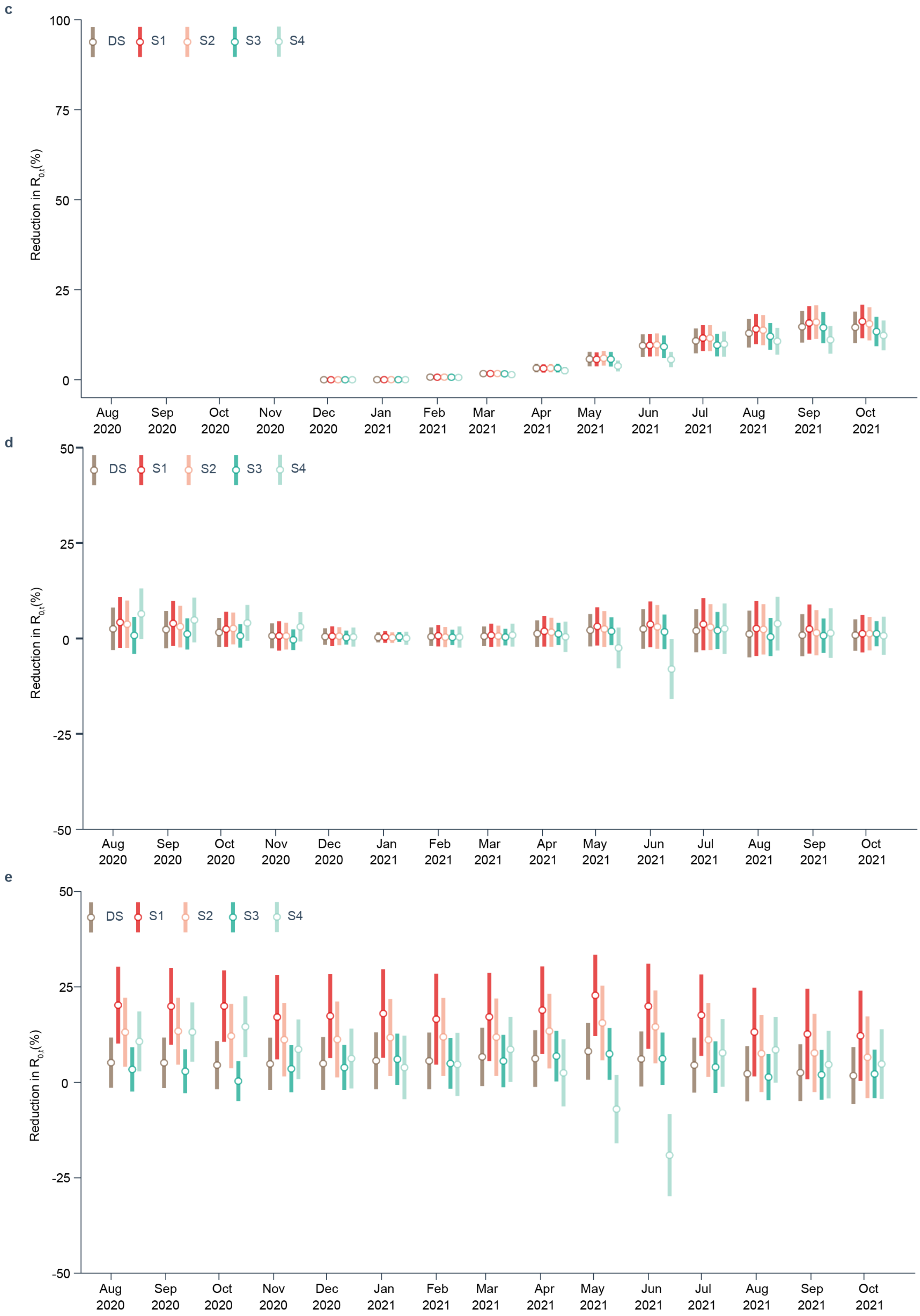

Supplementary Fig. 13 (Continue).

C. Model validation

C1. Prior and posterior predictive check

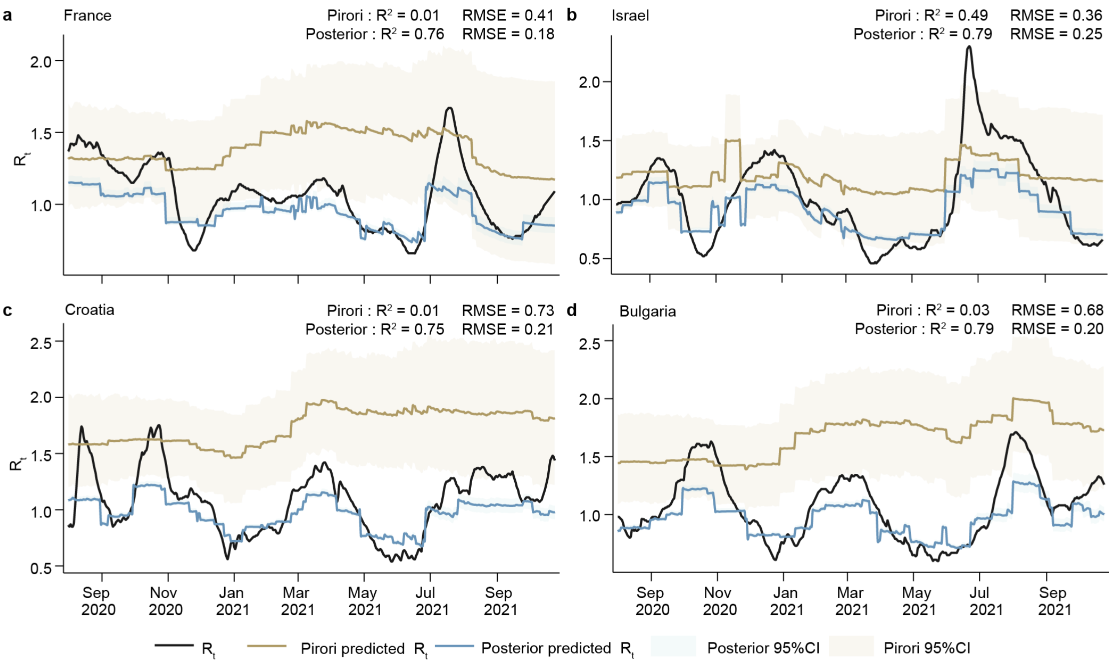

Supplementary Fig. 14 Comparison between our prior and posterior over the coefficients in our model for their predictability. Here, we take four countries with varied vaccination coverage (France 70%, Israel 66%, Croatia 43% and Bulgaria 21%) as examples. We first used our assumed prior to produce estimates of R_t by our model for the four countries, and then used the fitted model with posterior to generate another set of R_t for comparison. We repeated the above procedure for 10000 times and drawn the mean values and 95% confidence interval for demonstration.

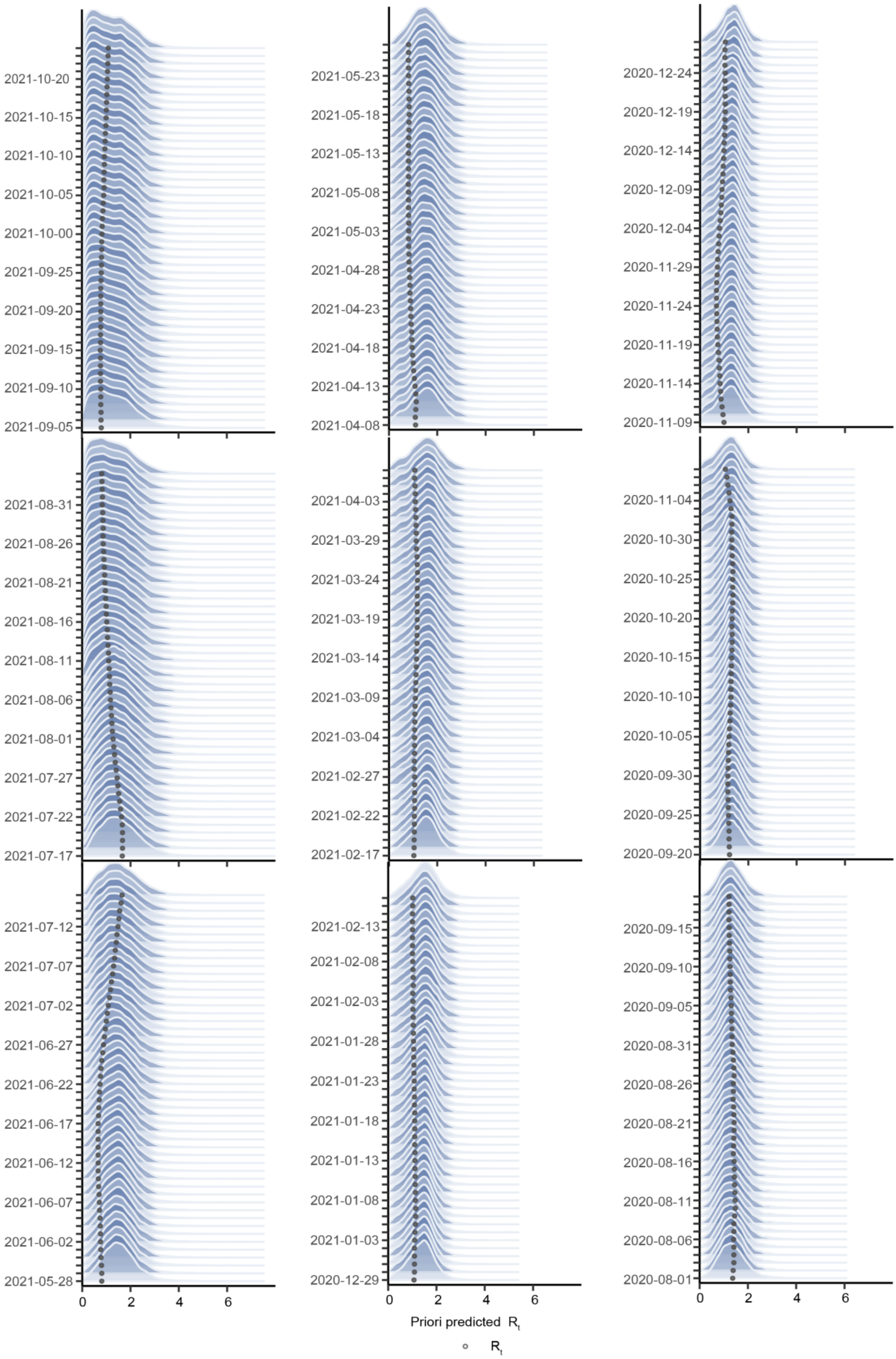

Supplementary Fig. 15 The prior predictive distribution in France. The time arrow line is from right to left and from bottom to top. The first day, 1/8/2020, is shown at the bottom right. The observed R_t values are illustrated by dots.

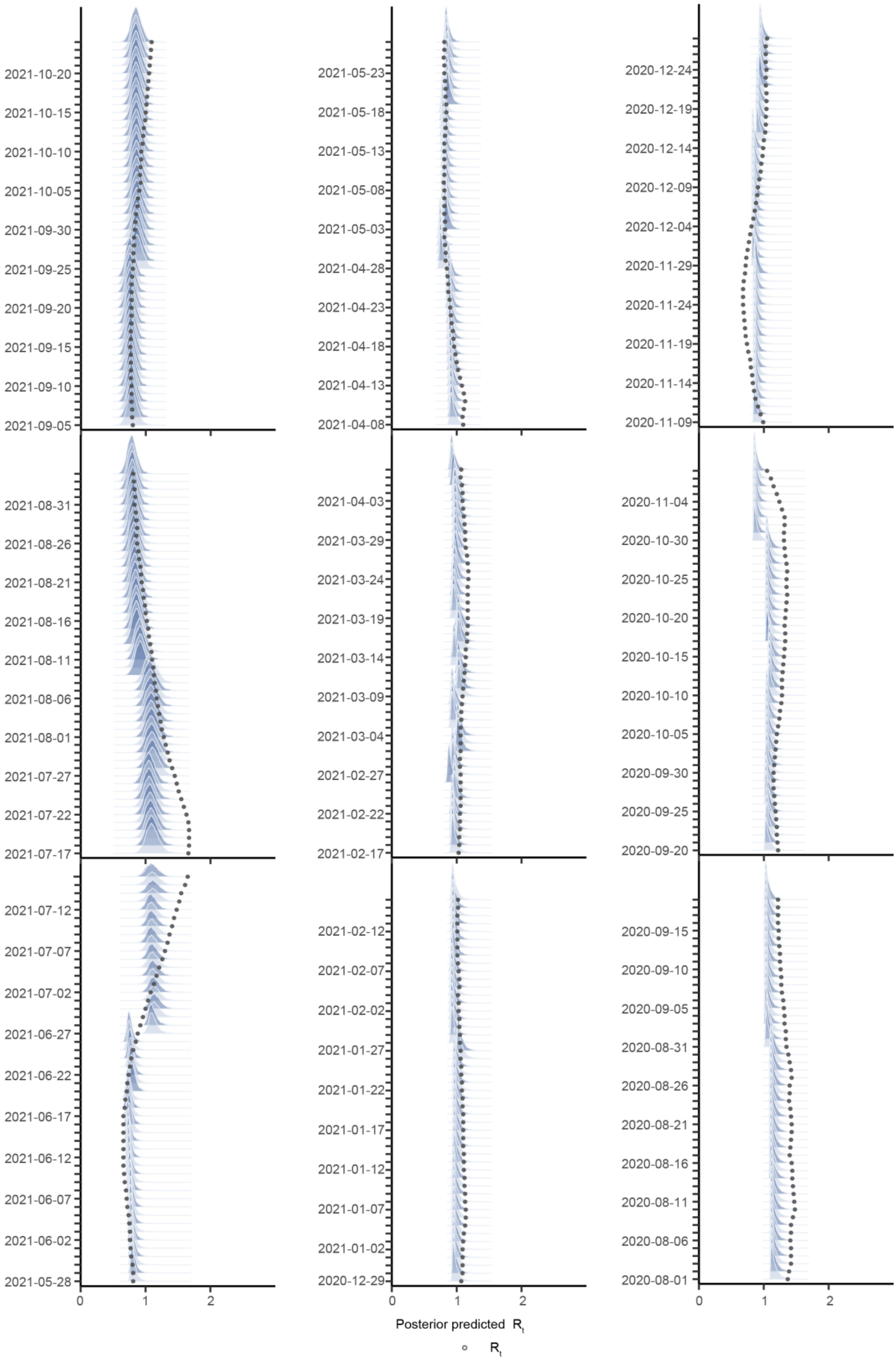

Supplementary Fig. 16 The posterior predictive distribution in France. The time arrow line is from right to left and from bottom to top. The first day, 1/8/2020, is shown at the bottom right. The observed R_t values are illustrated by dots.

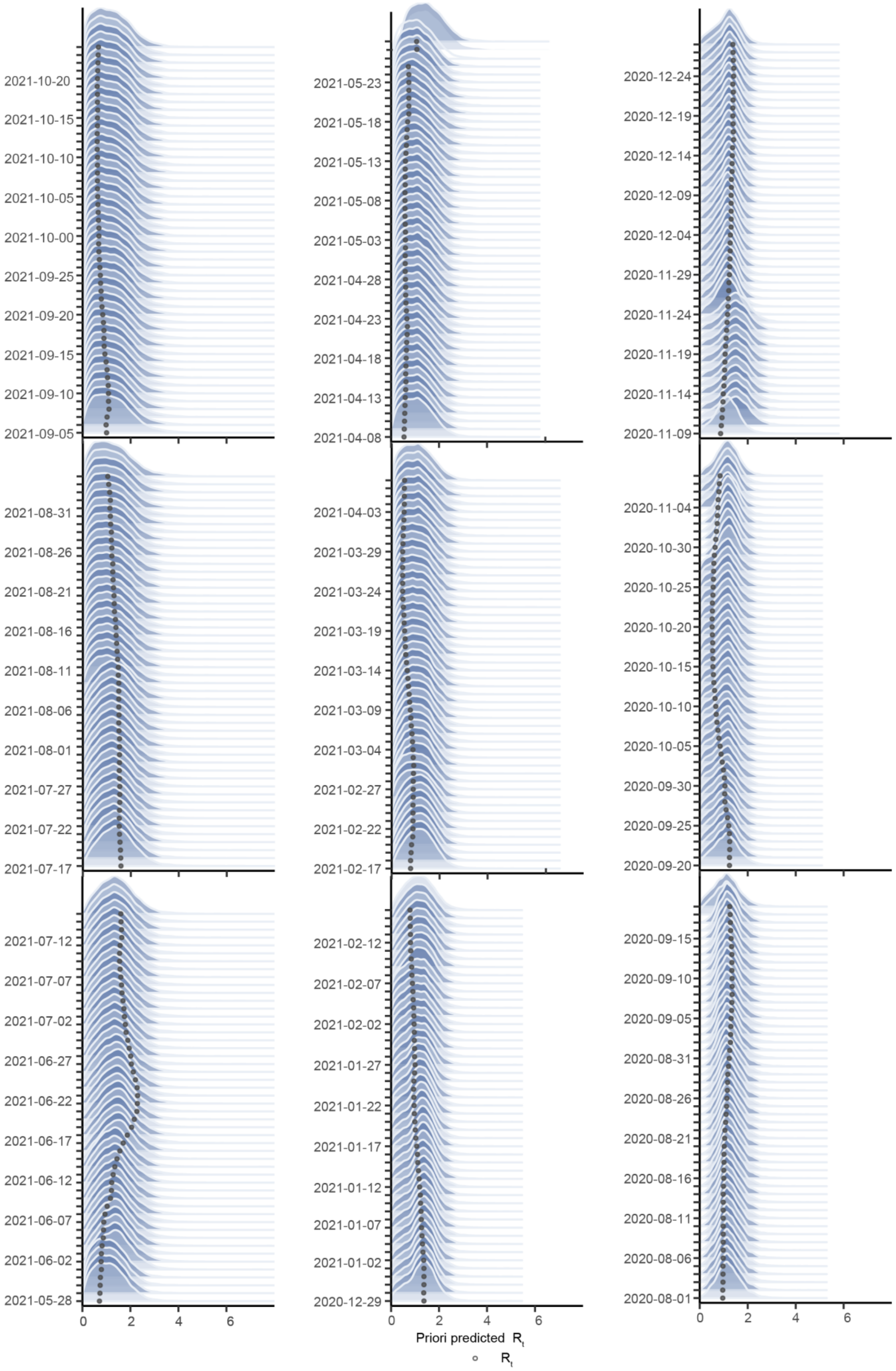

Supplementary Fig. 17 The prior predictive distribution in Israel. The time arrow line is from right to left and from bottom to top. The first day, 1/8/2020, is shown at the bottom right. The observed R_t values are illustrated by dots.

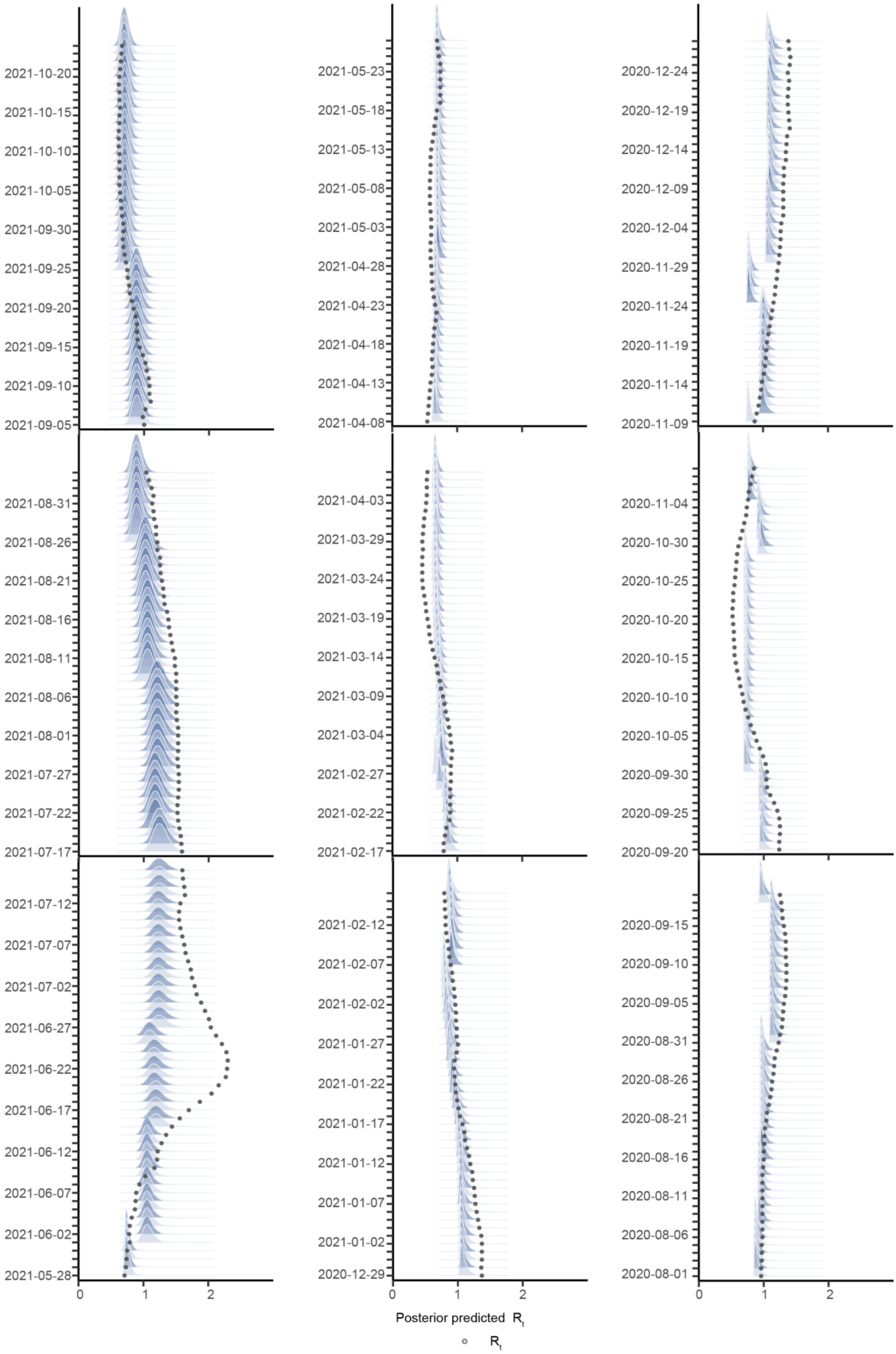

Supplementary Fig. 18 The posterior predictive distribution in Israel. The time arrow line is from right to left and from bottom to top. The first day, 1/8/2020, is shown at the bottom right. The observed R_t values are illustrated by dots.

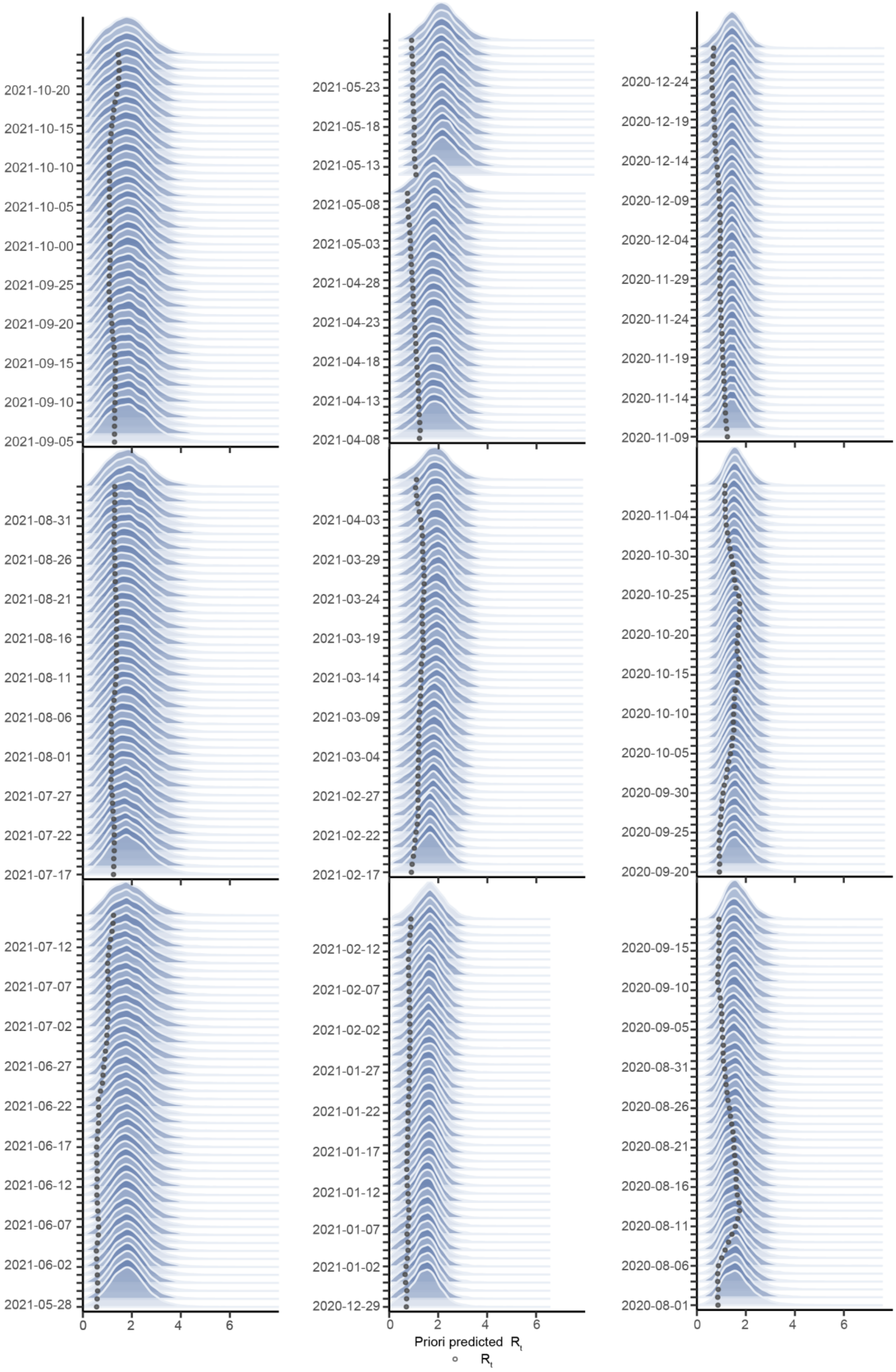

Supplementary Fig. 19 The prior predictive distribution in Croatia. The time arrow line is from right to left and from bottom to top. The first day, 1/8/2020, is shown at the bottom right. The observed R_t values are illustrated by dots.

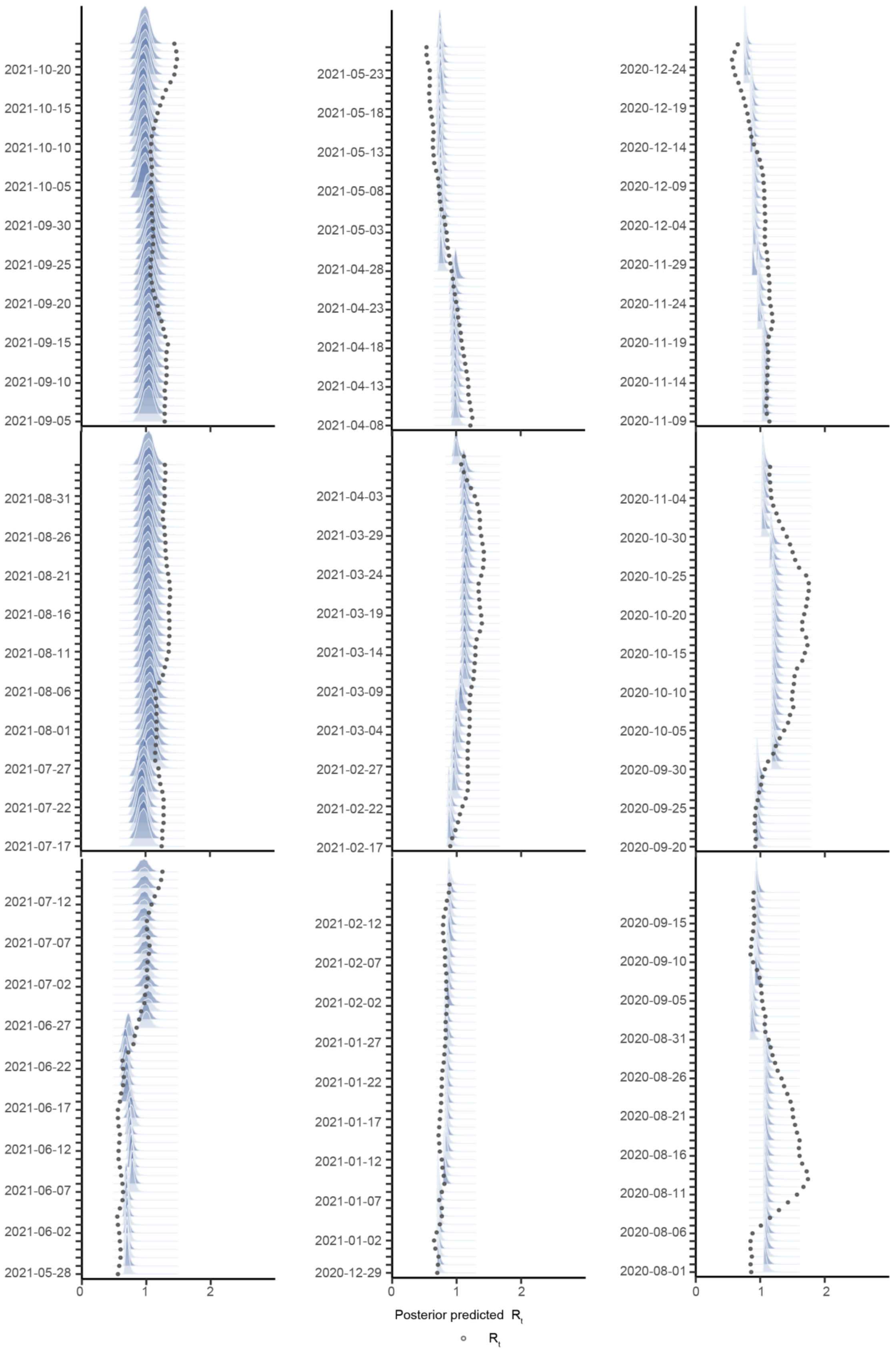

Supplementary Fig. 20 The posterior predictive distribution in Croatia. The time arrow line is from right to left and from bottom to top. The first day, 1/8/2020, is shown at the bottom right. The observed R_t values are illustrated by dots.

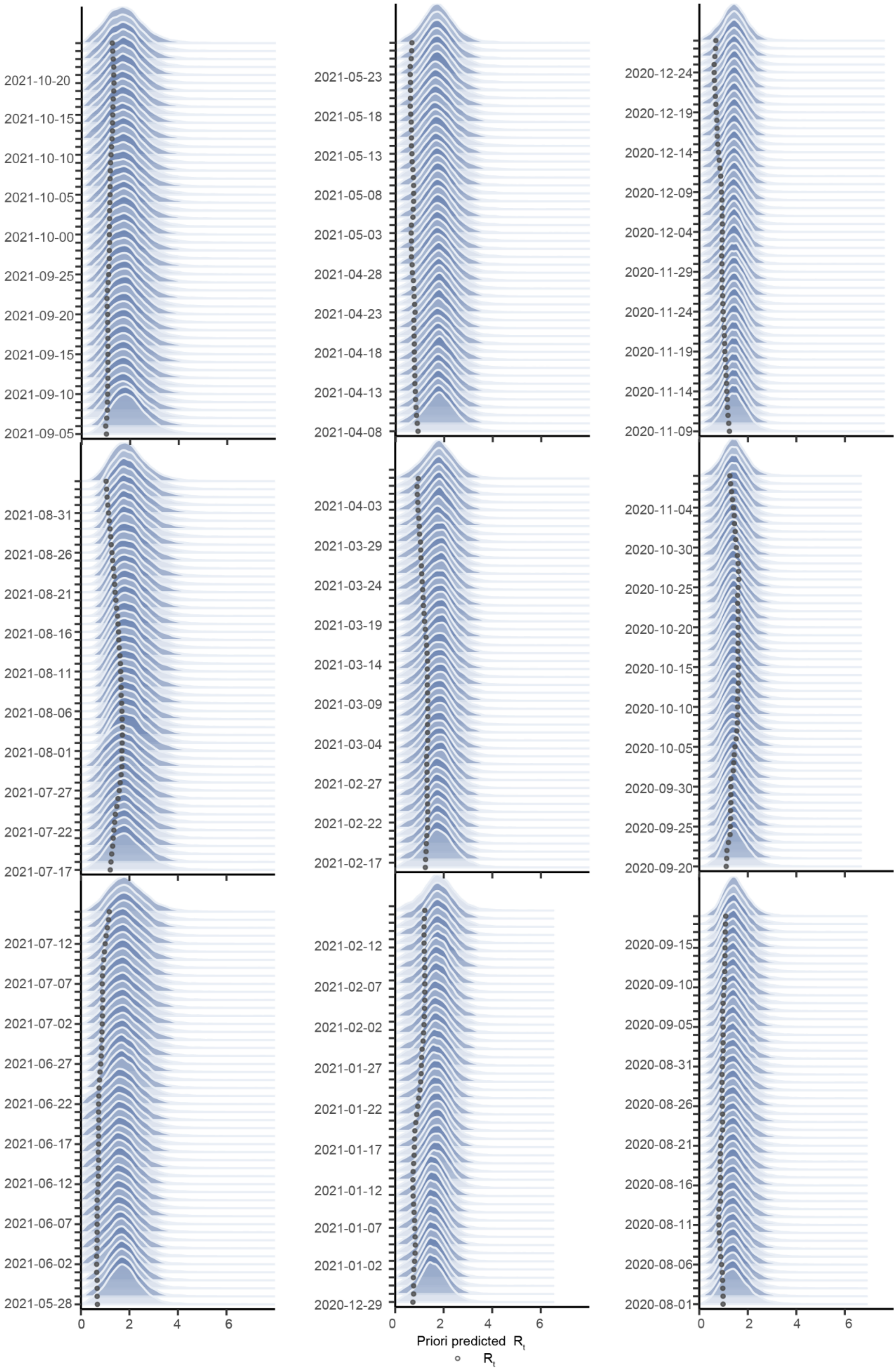

Supplementary Fig. 21 The prior predictive distribution in Bulgaria. The time arrow line is from right to left and from bottom to top. The first day, 1/8/2020, is shown at the bottom right. The observed R_t values are illustrated by dots.

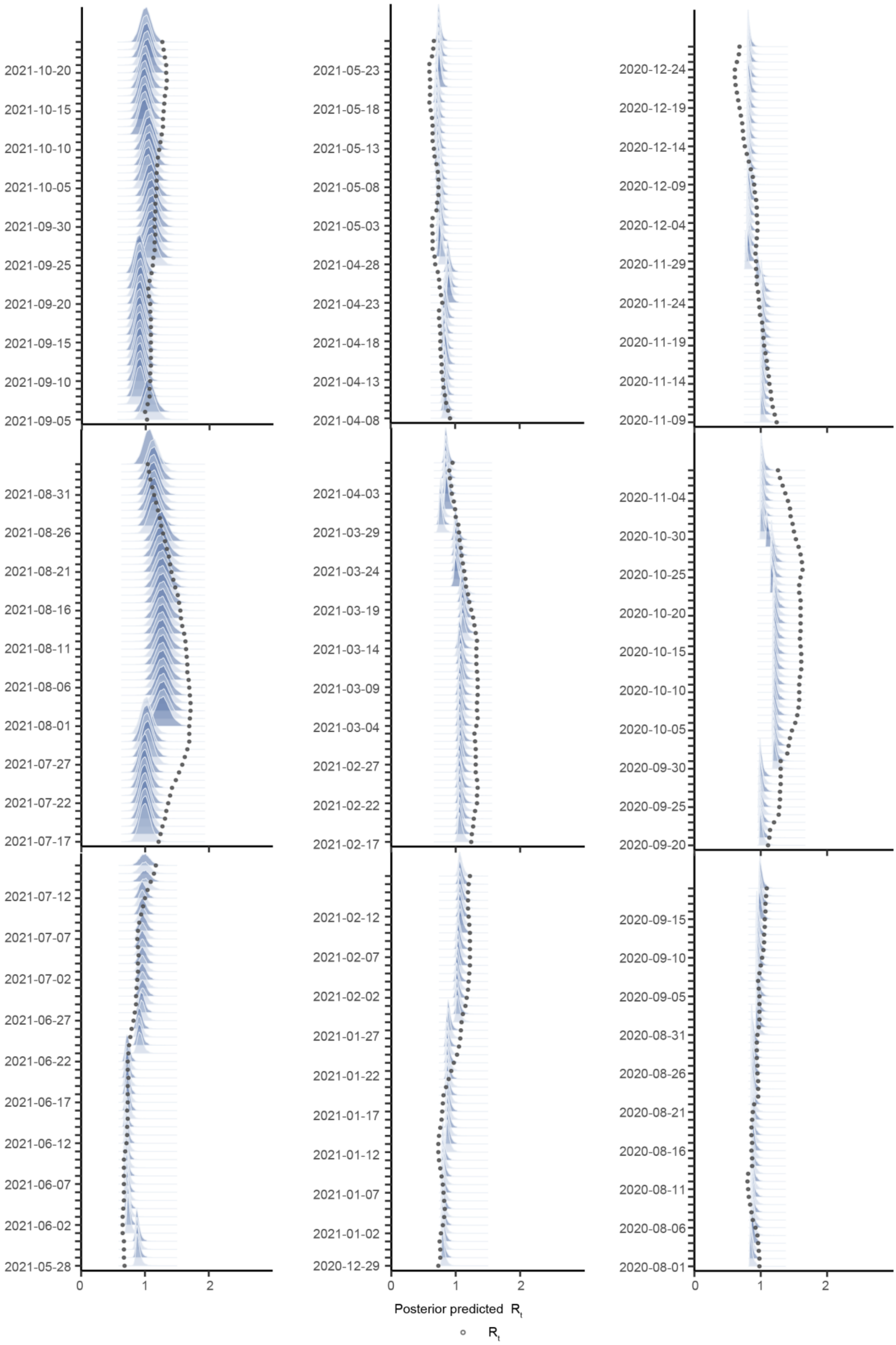

Supplementary Fig. 22 The posterior predictive distribution in Bulgaria. The time arrow line is from right to left and from bottom to top. The first day, 1/8/2020, is shown at the bottom right. The observed R_t values are illustrated by dots.

C2. MCMC convergence

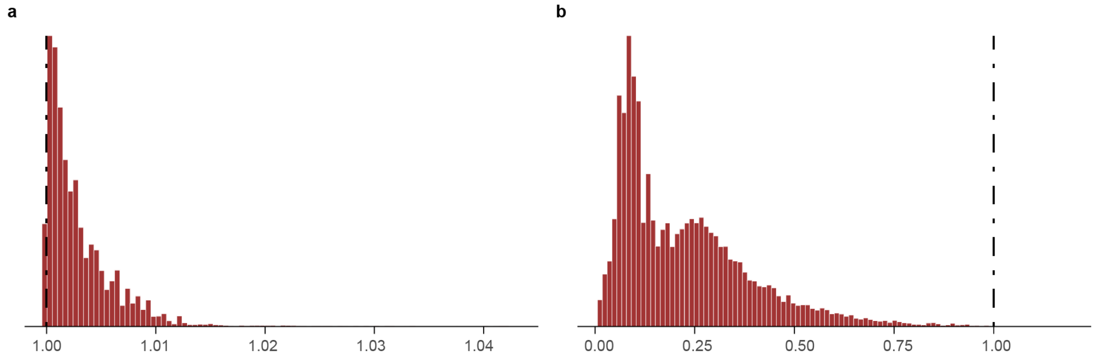

Supplementary Fig. 23 The convergence of MCMC in our estimation. (a)R-hat statistic taken from a run using the default model with default settings and values for all parameters. Values are close to 1, indicating convergence; (b)Relative effective sample size taken from a run using the default model with default settings and values for all parameters. Value 1 indicates perfect decorrelation between samples. Values above (below) 1 indicate that the effective number of samples is higher (lower) than the actual number of samples due to negative (positive) correlation, respectively.

C3. Meta-analysis

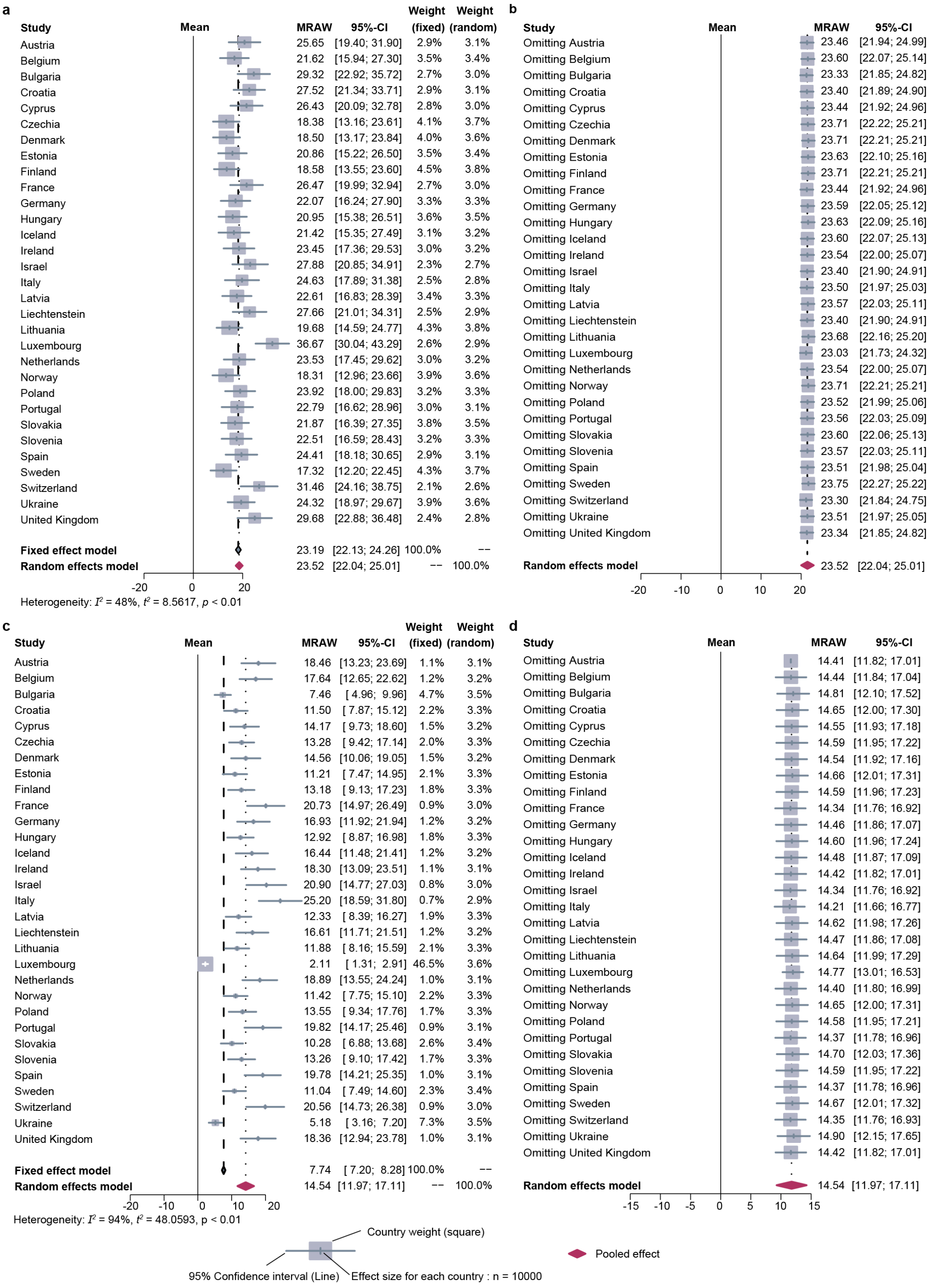

Supplementary Fig. 24 Forest plot comparing effects of NPIs and vaccination across 31 countries in October 2021, respectively. (a) Forest plot depicting the meta-analytical results for NPIs among 31 countries. (b) Forest plot depicting an influence analysis for NPIs omitting each country. (c) Forest plot depicting the meta-analytical results for vaccination among 31 countries. (d) Forest plot depicting an influence analysis for vaccination omitting each country. Noting the high heterogeneity among 31 countries, a random effects method was used to meta-analyse the data for each month.

C4. Leave-one-out cross validation

Pooling the national results using joint inference for each country

We used the leave-one-out method to validate our model. In each validation, 30 countries were used to pool the national results into the general European case. Then, the merged estimates were used to estimate the real-time R_t for the one country left. The model performance was evaluated by the difference between the predicted R_t and the observed values with the root mean square error (RMSE) and R-squared. In general, RMSE ranged from 0 to infinite with 0 representing the perfect prediction ability. R-squared was calculated by

$$R^2 = SS_{reg}/(SS_{reg} + SS_{res}),$$

$$SS_{reg} = \sum_i (f_i - \bar{y})^2,$$

$$SS_{res} = \sum_i (y_i - f_i)^2.$$

where f_i is the regressed value, and y_i is the observed value.

We repeated this process 31 times independently for each of the 31 study countries (see Supplementary Fig. 25). The median RMSE was 0.26 (IQR: 0.24 – 0.33) and R-squared ranged from 0.35 (Poland with vaccination rate 52%) to 0.76 (Ukraine with vaccination rate 0.16). Additionally, we also selected four countries with different levels of fully vaccination rate to illustrate the generalizability of our model (see Supplementary Fig. 26). The performance on explaining the variation of COVID-19 transmission for each country can be found in Supplementary Table 5.

Pooling the national data using a full model across countries

In contrast to pooling the national results by meta-analysis, the alternative approach to counter the heterogeneity across countries was to use all the data across countries to fit a single model. In comparison to the meta model, we also illustrate the leave-one-out

validation results for the four countries in Supplementary Fig. 27. Pooling the national results appears to be better explain power of the variation in R_t, where R-squared for France, Israel, Croatia, and Bulgaria is 0.51, 0.53, 0.37, and 0.38 for pooling the national data using a full model, respectively (Supplementary Fig. 28). Regarding R-squared, overall, pooling the national results improved 16% explanation power of pooling the national data using a full model across all countries. Moreover, by comparing Supplementary Fig. 25 and Supplementary Fig. 27, we can find that pooling national data too flattened the heterogeneity across countries than pooling the national results, leading the predicted R_t to an average level of all countries but losing the characteristics of each specific country.

Supplementary Table 5. R-squared for the leave-one-out cross validation across countries. Fully vaccination rate was reported by 25 October 2021.

Country	R^2	Fully vaccination rate (%)	Country	R^2	Fully vaccination rate (%)
Austria	0.57	64	Iceland	0.75	81
Belgium	0.61	74	Israel	0.62	66
Bulgaria	0.43	21	Italy	0.61	86
Switzerland	0.40	63	Liechtenstein	0.55	62
Cyprus	0.42	47	Lithuania	0.37	60
Czechia	0.44	57	Luxembourg	0.69	6
Germany	0.70	67	Latvia	0.40	52
Denmark	0.46	76	Netherlands	0.46	73
Spain	0.64	80	Norway	0.46	68
Estonia	0.49	58	Poland	0.35	52
Finland	0.64	68	Portugal	0.70	89
France	0.63	70	Slovakia	0.44	42
United Kingdom	0.58	68	Slovenia	0.49	53
Croatia	0.44	43	Sweden	0.46	68
Hungary	0.45	59	Ukraine	0.76	16
Ireland	0.60	77			

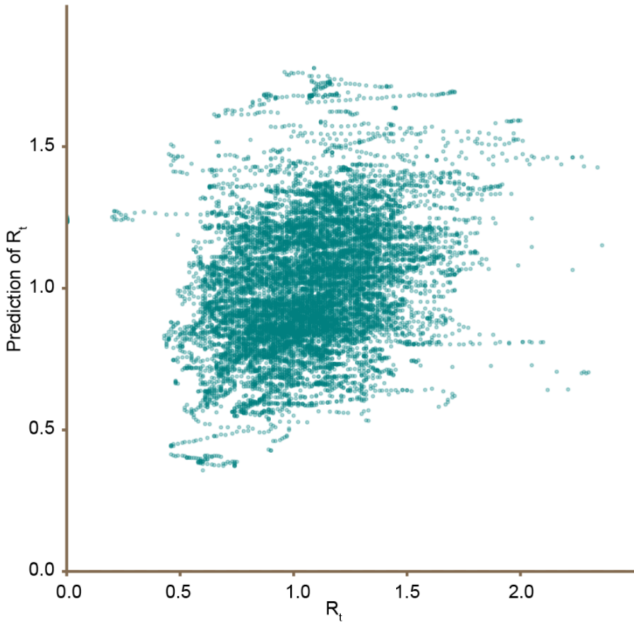

Supplementary Fig. 25 The results of leave-one-out-validation over 31 study countries generated by pooling the national results. The overall r-squared is 0.55.

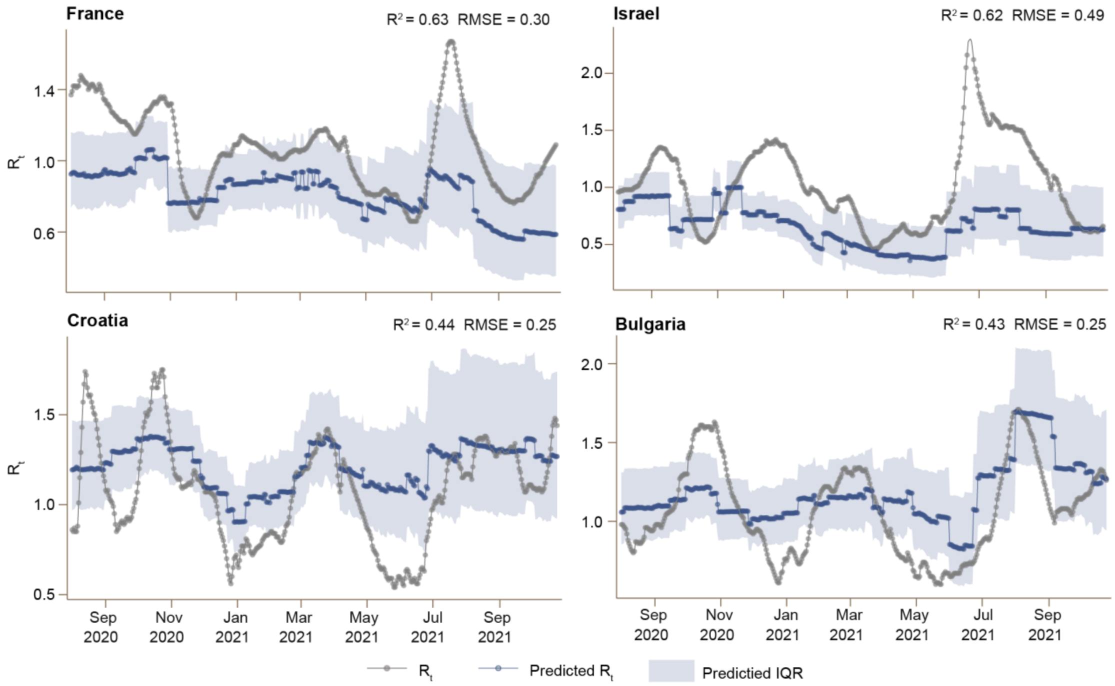

Supplementary Fig. 26 Comparison between the predicted real-time R_t and the empirical real-time R_t by pooling the national results. The predicted R_t were represented by blue dots (median) and bands (interquartile range), where the empirical R_t were represented by grey dots. Here, we take four countries with varied vaccination coverage (France 70%, Israel 66%, Croatia 43% and Bulgaria 21%) as examples.

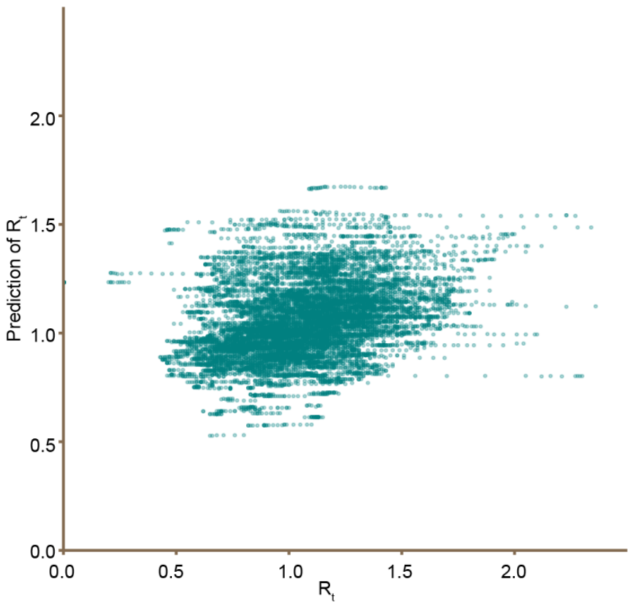

Supplementary Fig. 27 The results of leave-one-out-validation over 31 study countries generated by pooling the national data using a full model. The overall r-squared is 0.50.

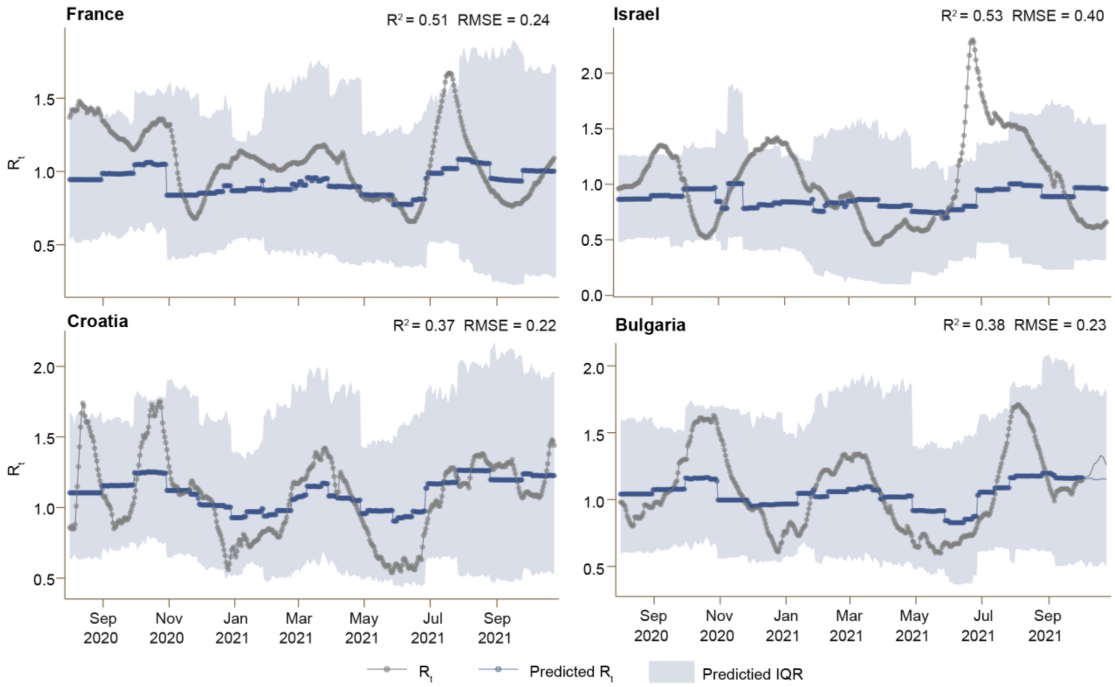

Supplementary Fig. 28 Comparison between the predicted real-time R_t and the empirical real-time R_t by pooling the national data to fit a single model. The predicted R_t were represented by blue dots (median) and bands (interquartile range), where the empirical R_t were represented by grey dots. Here, we take four countries with varied vaccination coverage (France 70%, Israel 66%, Croatia 43% and Bulgaria 21%) as examples.

D. Additional discussions

D1. Limitations of using reproduction number

We used the reproduction number to characterise the changes in COVID-19 transmission dynamics and disentangle the impact of NPIs and vaccination. Here, we give an additional discussion on the limitation of using the reproduction number, which can be estimated by many different mathematical models. As R_t can hardly get observed directly, it is usually calculated via a mathematical model. However, different methods can give a different estimate of R_t, which may limit its accuracy and usefulness[24].

Besides, R_0 cannot be modified through vaccination or other changes in population susceptibility, but it can vary based on several biological, socio-behavioural, and environmental factors[25]. It can also be modified by physical distancing and other public policy or social interventions, although some historical definitions exclude any deliberate intervention in reducing disease transmission, including non-pharmacological interventions. And indeed, whether NPIs are included in R_0 often depends on the paper, disease, and what if any intervention is being studied. This creates some confusion, because R_0 is not a constant, whereas most mathematical parameters with "nought" subscripts are constants. In our study, we used the R_0 of SARS-CoV-2 estimated by the highest R_t after the large-scale relaxation of NPIs but before VOCs became predominant in communities, representing the approximate transmissibility without interventions, and R_t was used to represent the transmission variation over time, under various settings and the circulation of different SARS-CoV-2 variants.

D2. Using stringency index of NPIs

Of note, we used the stringency index produced by the OxCGRT[26] to represent the government COVID-19 response level in terms of ordinal containment, closure policy and public information campaigns, which is calculated by the strictness of ‘lockdown style’ policies that primarily restrict people’s behaviour including school closures, workplace closures, and travel bans. OxCGRT calculated the stringency index as a composite measure of nine of the response metrics, including 1) school closures; 2) workplace closures; 3) cancellation of public events; 4) restrictions on public gatherings; 5) closures of public transport; 6) stay-at-home requirements; 7) public information campaigns; 8) restrictions on internal movements; and 9) international travel controls. OxCGRT collected the nine response metrics using different scores in their database, to measure the strength of policies. To calculate the stringency index, the nine metrics were first normalised to a value between 0 and 100 by an additional flag variable representing whether they are "targeted" to a specific geographical region (flag=0) or whether they are a "general" policy that is applied across the whole country/territory (flag=1). That is,

$$I_{i,t} = 100(m_{i,t} - 0.5(1 - f_{i,t}))/M_i$$

where $I_{i,t}$ was the normalised value of metric i at day t, $m_{i,t}$ was the score value of metric metric i at day t, $f_{i,t}$ was the flag of metric i at day t, and M_i was the highest score defined for the metric i. Then, the stringency index of a day was defined as the average value of the normalised nine metrics at that day in each country. A higher score indicates a stricter response (i.e., 100 = strictest response). If policies vary at the subnational level, the index is shown as the response level of the strictest sub-region. In the model of this study, the stringency index has been normalised by min-max normalisation, ranging from 0 to 1.

As heterogeneous countries may enact different packages of NPIs and the same intervention may also be implemented in different ways across neighbouring countries[27], it is hard to estimate the standard effect and criteria of NPIs that apply to all countries. In contrast, we used the relative common concept of the response level against COVID-19 transmission. For the same strength of the response, different countries are allowed to deploy NPIs with different combinations. Haug et al.[28] documented that less disruptive and costly NPIs can be as effective as more intrusive, drastic ones (for example, a national lockdown) in March–April 2020, thus a suitable combination of NPIs is necessary to curb the spread of the virus. In fact, strong associations between the OxCGRT's stringency index and mobility metrics have been evidenced[26]. Even though the effect of individual NPIs cannot be estimated in this way, our study still provided insights on the response level that each country should have, to control COVID-19 in the vaccination era.

D3. Herd immunity threshold

The one of most important uses of R0 is to determine what percentage of the population should be immunised through vaccination to fully contain a disease, i.e. reducing the reproduction number below 1. Generally, the larger the value of R0, the harder it is to control the epidemic. For simple models, the proportion of the population that needs to be effectively immunised (meaning not susceptible to infection) to prevent sustained spread of the disease must be larger than $1\text{-}1/R0$[29]. Conversely, the proportion of the population that remains susceptible to infection in the endemic equilibrium is $1/R0$. Supplementary Fig. 29 shows the thresholds of herd immunity in terms of SARS-CoV-2 and its variants.

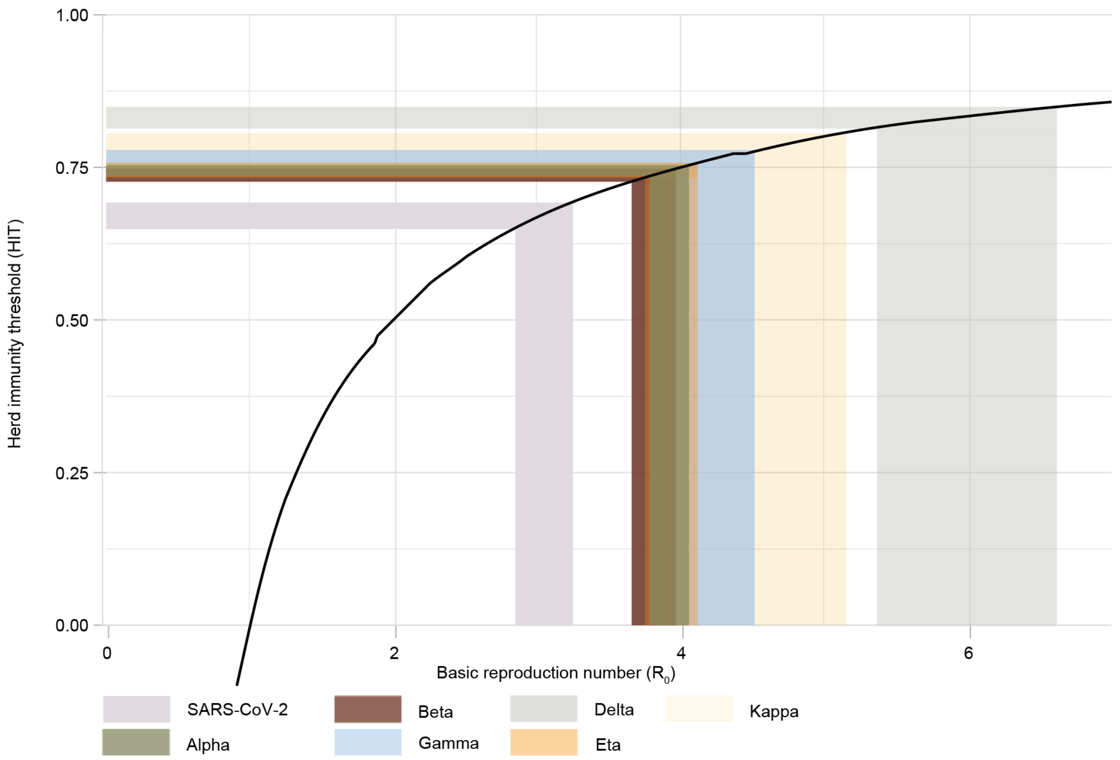

Supplementary Fig. 29 The thresholds of herd immunity in terms of SARS-CoV-2 and its variants. Of note, the threshold here refers to the fraction of the population that should be immune instead of the population that has been vaccinated.

Reference

1. Arroyo-Marioli F, Bullano F, Kucinskas S, Rondón-Moreno C (2021) Tracking R of COVID-19: A new real-time estimation using the Kalman filter. PLoS ONE 16(1): e0244474. https://doi.org/10.1371/journal.pone.0244474
2. Fraser, C., Donnelly, C.A., Cauchemez, S., Hanage, W.P., Van Kerkhove, M.D., Hollingsworth, T.D., Griffin, J., Baggaley, R.F., Jenkins, H.E., Lyons, E.J. and Jombart, T., 2009. Pandemic potential of a strain of influenza A (H1N1): early findings. science, 324(5934), pp.1557-1561.
3. Curran, J., Dol, J., Boulos, L., Somerville, M., & McCulloch, H. Public Health and Health Systems Impacts of SARS-CoV-2 Variants of Concern.
4. US Centers for Disease Control and Prevention. SARS-CoV-2 variant classifications and definitions. Updated Aug 2021. https://www.cdc.gov/coronavirus/2019-ncov/variants/variant-info.html
5. Campbell, F., Archer, B., Laurenson-Schafer, H., Jinnai, Y., Konings, F., Batra, N., Pavlin, B., Vandemaele, K., Van Kerkhove, M.D., Jombart, T. and Morgan, O., 2021. Increased transmissibility and global spread of SARS-CoV-2 variants of concern as at June 2021. Eurosurveillance, 26(24), p.2100509.
6. Petherick, A., Goldszmidt, R., Andrade, E.B., Furst, R., Hale, T., Pott, A. and Wood, A., 2021. A worldwide assessment of changes in adherence to COVID-19 protective

behaviours and hypothesized pandemic fatigue. Nature Human Behaviour, 5(9), pp.1145-1160.

7. Lai, S., Ruktanonchai, N.W., Carioli, A., Ruktanonchai, C.W., Floyd, J.R., Prosper, O., Zhang, C., Du, X., Yang, W. and Tatem, A.J., 2021. Assessing the Effect of Global Travel and Contact Restrictions on Mitigating the COVID-19 Pandemic. Engineering.
8. Locatelli, I., Trächsel, B. and Rousson, V., 2021. Estimating the basic reproduction number for COVID-19 in Western Europe. Plos one, 16(3), p.e0248731.
9. Al Kaabi, N., Zhang, Y., Xia, S., Yang, Y., Al Qahtani, M.M., Abdulrazzaq, N., Al Nusair, M., Hassany, M., Jawad, J.S., Abdalla, J. and Hussein, S.E., 2021. Effect of 2 Inactivated SARS-CoV-2 Vaccines on Symptomatic COVID-19 Infection in Adults: A Randomized Clinical Trial. Jama.
10. Logunov, D.Y., Dolzhikova, I.V., Zubkova, O.V., Tukhvatullin, A.I., Shcheblyakov, D.V., Dzharullaeva, A.S., Grousova, D.M., Erokhova, A.S., Kovyrshina, A.V., Botikov, A.G. and Izhaeva, F.M., 2020. Safety and immunogenicity of an rAd26 and rAd5 vector-based heterologous prime-boost COVID-19 vaccine in two formulations: two open, non-randomised phase 1/2 studies from Russia. The Lancet, 396(10255), pp.887-897.
11. Polack, F.P., Thomas, S.J., Kitchin, N., Absalon, J., Gurtman, A., Lockhart, S., Perez, J.L., Marc, G.P., Moreira, E.D., Zerbini, C. and Bailey, R., 2020. Safety and efficacy of the BNT162b2 mRNA Covid-19 vaccine. *New England Journal of Medicine*.
12. US FDA. Janssen Ad26.COV2.S (COVID-19. Vaccine VRBPAC Briefing Document. 26 févr 2020 775[cité 29 mars 2021]; Disponible sur: https://www.fda.gov/media/146217/dow
13. Voysey, M., Clemens, S.A.C., Madhi, S.A., Weckx, L.Y., Folegatti, P.M., Aley, P.K., Angus, B., Baillie, V.L., Barnabas, S.L., Bhorat, Q.E. and Bibi, S., 2021. Single-dose administration and the influence of the timing of the booster dose on immunogenicity and efficacy of ChAdOx1 nCoV-19 (AZD1222) vaccine: a pooled analysis of four randomised trials. The Lancet, 397(10277), pp.881-891.
14. Baden, L. R. et al. Efficacy and safety of the mRNA-1273 SARS-CoV-2 vaccine. N. Engl. J. Med. 384, 403–416 (2021).
15. https://apps.who.int/iris/bitstream/handle/10665/331773/WHO-2019-nCoV-Adjusting_PH_measures-2020.1-eng.pdf
16. https://www.ecdc.europa.eu/en/publications-data/download-todays-data-geographic-distribution-covid-19-cases-worldwide
17. https://github.com/CSSEGISandData/COVID-19
18. https://raw.githubusercontent.com/owid/covid-19-data/master/public/data/testing/covid-testing-all-observations.csv
19. https://covid19.apple.com/mobility
20. https://www.google.com/covid19/mobility/
21. Chowell, G., Hengartner, N.W., Castillo-Chavez, C., Fenimore, P.W. and Hyman, J.M., 2004. The basic reproductive number of Ebola and the effects of public health measures: the cases of Congo and Uganda. Journal of theoretical biology, 229(1), pp.119-126.
22. Ajelli, M., Iannelli, M., Manfredi, P. and degli Atti, M.L.C., 2008. Basic mathematical models for the temporal dynamics of HAV in medium-endemicity Italian areas. Vaccine, 26(13), pp.1697-1707.
23. Heffernan, J.M., Smith, R.J. and Wahl, L.M., 2005. Perspectives on the basic reproductive ratio. Journal of the Royal Society Interface, 2(4), pp.281-293.

24. Li, J. and Blakeley, D., The failure of $R0$. Computational and Mathematical Methods in Medicine, 2011.
25. Delamater, P.L., Street, E.J., Leslie, T.F., Yang, Y.T. and Jacobsen, K.H., 2019. Complexity of the basic reproduction number (R0). Emerging infectious diseases, 25(1), p.1.
26. Thomas Hale, Noam Angrist, Rafael Goldszmidt, Beatriz Kira, Anna Petherick, Toby Phillips, Samuel Webster, Emily Cameron-Blake, Laura Hallas, Saptarshi Majumdar, and Helen Tatlow. (2021). "A global panel database of pandemic policies (Oxford COVID-19 Government Response Tracker)." Nature Human Behaviour. https://doi.org/10.1038/s41562-021-01079-8
27. Ruktanonchai, N.W., Floyd, J.R., Lai, S., Ruktanonchai, C.W., Sadilek, A., Rente-Lourenco, P., Ben, X., Carioli, A., Gwinn, J., Steele, J.E. and Prosper, O., 2020. Assessing the impact of coordinated COVID-19 exit strategies across Europe. Science, 369(6510), pp.1465-1470.
28. Haug, N., Geyrhofer, L., Londei, A. et al. Ranking the effect of worldwide COVID-19 government interventions. Nat Hum Behav 4, 1303–1312 (2020). https://doi.org/10.1038/s41562-020-01009-0
29. Fine, P., Eames, K. and Heymann, D.L., 2011. "Herd immunity": a rough guide. Clinical infectious diseases, 52(7), pp.911-916.
