# Supplementary material for: Untangling the changing impact of non-pharmaceutical interventions and vaccination on European COVID-19 trajectories
Source: Nat Commun. 2022 Jun 3;13:3106. doi: 10.1038/s41467-022-30897-1 (PMC9166696; doi:10.1038/s41467-022-30897-1)
Supplement: Supplementary file 3 — Reporting Summary [file 41467_2022_30897_MOESM3_ESM.pdf]

nature portfolio

Corresponding author(s): Yong Ge & Shengjie Lai & Andrew J Tatem

Last updated by author(s): May 9, 2022

Reporting Summary

Nature Portfolio wishes to improve the reproducibility of the work that we publish. This form provides structure for consistency and transparency in reporting. For further information on Nature Portfolio policies, see our Editorial Policies and the Editorial Policy Checklist.

Statistics

For all statistical analyses, confirm that the following items are present in the figure legend, table legend, main text, or Methods section.

n/a	Confirmed	
☐	☒	The exact sample size (n) for each experimental group/condition, given as a discrete number and unit of measurement
☐	☒	A statement on whether measurements were taken from distinct samples or whether the same sample was measured repeatedly
☐	☒	The statistical test(s) used AND whether they are one- or two-sided<br>*Only common tests should be described solely by name; describe more complex techniques in the Methods section.*
☐	☒	A description of all covariates tested
☐	☒	A description of any assumptions or corrections, such as tests of normality and adjustment for multiple comparisons
☐	☒	A full description of the statistical parameters including central tendency (e.g. means) or other basic estimates (e.g. regression coefficient) AND variation (e.g. standard deviation) or associated estimates of uncertainty (e.g. confidence intervals)
☐	☒	For null hypothesis testing, the test statistic (e.g. F, t, r) with confidence intervals, effect sizes, degrees of freedom and P value noted<br>*Give P values as exact values whenever suitable.*
☐	☒	For Bayesian analysis, information on the choice of priors and Markov chain Monte Carlo settings
☒	☐	For hierarchical and complex designs, identification of the appropriate level for tests and full reporting of outcomes
☐	☒	Estimates of effect sizes (e.g. Cohen's d, Pearson's r), indicating how they were calculated

Our web collection on statistics for biologists contains articles on many of the points above.

Software and code

Policy information about availability of computer code

Data collection	R version 4.1.1 (R Foundation for Statistical Computing, Vienna, Austria) was used to perform data collation and analyses.
Data analysis	R version 4.1.1 (R Foundation for Statistical Computing, Vienna, Austria) was used to perform data collation and analyses. The R packages used in this study for data analysis are ‘meta’ with version 5.2-0 and ‘rstan’ with version 2.21.5. The model built by this study has been made openly available for further use at https://github.com/wxl1379457192/Vaccine-NPIs-in-EuropeV2.

For manuscripts utilizing custom algorithms or software that are central to the research but not yet described in published literature, software must be made available to editors and reviewers. We strongly encourage code deposition in a community repository (e.g. GitHub). See the Nature Portfolio guidelines for submitting code & software for further information.

Data

Policy information about availability of data

All manuscripts must include a data availability statement. This statement should provide the following information, where applicable:

- Accession codes, unique identifiers, or web links for publicly available datasets
- A description of any restrictions on data availability
- For clinical datasets or third party data, please ensure that the statement adheres to our policy

The data used in this study are publicly available online at https://github.com/owid/covid-19-data/tree/master/public/data. The processed climate data are available online at https://github.com/wxl1379457192/Vaccine-NPIs-in-EuropeV2.

March 2021

Field-specific reporting

Please select the one below that is the best fit for your research. If you are not sure, read the appropriate sections before making your selection.

☐ Life sciences ☒ Behavioural & social sciences ☐ Ecological, evolutionary & environmental sciences

For a reference copy of the document with all sections, see nature.com/documents/nr-reporting-summary-flat.pdf

Behavioural & social sciences study design

All studies must disclose on these points even when the disclosure is negative.

Study description	Quantitative observational and modelling study
Research sample	Reproduction rate, timing and intensity of non-pharmaceutical interventions (stringency index), variant structure and vaccinated population reported across 31 countries as of October 25, 2021, and country-specific characteristics during the corresponding period for each country. The daily reproduction number (Rt) and full vaccination rate were collected from the websites of Our World in Data (https://ourworldindata.org/coronavirus-source-data). We also collected the national data (across 31 countries) of daily intensities of non-pharmaceutical interventions (NPIs) from Oxford Covid-19 Government Response Tracker (https://github.com/OxCGRT/covid-policy-tracker/tree/master/data). The biweekly variant structure data of SARS-CoV-2 and its related variants was collected between 21 December 2020 and 25 October 2021 from Global Initiative on Sharing All Influenza Data (GISAID) (https://www.gisaid.org/). The climate data was collated from the Global Land Data Assimilation System (https://developers.google.com/earth-engine/datasets/catalog/NASA_GLDAS_V021_NOAH_G025_T3H).
Sampling strategy	The number of datasets involved in this study was determined by the study object. In addition to the necessary reproduction rate, stringency index and fully vaccination rate datasets, variant structure dataset was used to explain the different COVID-19 transmission contexts across countries and the climate dataset was used to explain the different environmental conditions across countries.
Data collection	All the data used in this study are publicly available from data sources and processed in our manuscript by R. The researchers were not blinded to experimental condition.
Timing	Reproduction numbers: August 1, 2020 - September 23, 2021. Vaccination rate: August 1, 2020 - September 23, 2021. Non-pharmaceutical interventions: August 1, 2020 - October 25, 2021. Variant structure data: between 21 December 2020 and 25 October 2021. Humidity and air temperature: January 1, 2020 - October 25, 2021
Data exclusions	No data were excluded from the analyses.
Non-participation	No participants were involved in the study.
Randomization	The heterogeneity across different countries makes them um-comparable regarding the impact of NPIs and vaccination.

Reporting for specific materials, systems and methods

We require information from authors about some types of materials, experimental systems and methods used in many studies. Here, indicate whether each material, system or method listed is relevant to your study. If you are not sure if a list item applies to your research, read the appropriate section before selecting a response.

Materials & experimental systems

n/a	Involved in the study	
☒	☐	Antibodies
☒	☐	Eukaryotic cell lines
☒	☐	Palaeontology and archaeology
☒	☐	Animals and other organisms
☒	☐	Human research participants
☒	☐	Clinical data
☒	☐	Dual use research of concern

Methods

n/a	Involved in the study	
☒	☐	ChIP-seq
☒	☐	Flow cytometry
☒	☐	MRI-based neuroimaging
